# Photocatalytic Degradation of Naproxen: Intermediates and Total Reaction Mechanism

**DOI:** 10.3390/molecules29112583

**Published:** 2024-05-30

**Authors:** Daniela González-Pereyra, Ilse Acosta, Brenda Zermeño, Johana Aguilar, Elisa Leyva, Edgar Moctezuma

**Affiliations:** Facultad de Ciencias Químicas, Universidad Autónoma de San Luis Potosí, Av. Manuel Nava # 6, San Luis Potosí 78210, Mexico; danni.gper@gmail.com (D.G.-P.); acosta.m_ilse@hotmail.com (I.A.); brenda.zermeno@uaslp.mx (B.Z.); johana.aguilar@uaslp.mx (J.A.)

**Keywords:** naproxen, adsorption, photocatalytic degradation, TiO_2_, reaction mechanism

## Abstract

Photochemical and photocatalytic oxidation of naproxen (NPX) with UV-A light and commercial TiO_2_ under constant flow of oxygen have been investigated. Adsorption experiments indicated that 90% of the solute remained in the solution. Combined chemical analysis of samples on the photochemical degradation indicated that NPX in an aqueous solution (20 ppm) is efficiently transformed into other species but only 18% of the reactant is mineralized into CO_2_ and water after three hours of reaction. Performing the photocatalytic oxidation in the presence of TiO_2_, more than 80% of the organic compounds are mineralized by reactive oxidation species (ROS) within four hours of reaction. Analysis of reaction mixtures by a combination of analytical techniques indicated that naproxen is transformed into several aromatic naphthalene derivatives. These latter compounds are eventually transformed into polyhydroxylated aromatic compounds that are strongly adsorbed onto the TiO_2_ surface and are quickly oxidized into low-molecular-weight acids by an electron transfer mechanism. Based on this and previous studies on NPX photocatalytic oxidation, a unified and complete degradation mechanism is presented.

## 1. Introduction

Many water bodies around the word have been polluted with waste produced by industrial, agricultural, farming, hospital, and domestic activities. The vast majority of contaminants are synthetic organic chemical compounds such as surfactants, pesticides, pharmaceuticals, personal care products, illicit drugs, and stimulants [1,2,3,4,5,6,7,8]. Pharmaceuticals, also named medications, are chemical compounds used to treat diseases and to improve the quality of life of humans and farm animals. They have already been detected in wastewater, surface water, wells, marine sediments, and even in drinking water [1,2,4,9,10,11,12]. In Mexico, 39.7% of surface water bodies are contaminated as indicated by their high values of COD, BOD, TOC, and toxicity to Daphnia magna and Vibrio fischeri [13]. Among the compounds detected are residues of anti-inflammatories, analgesics, antibiotics, antipyretics, antidepressants, hormones, anesthetics, lipid regulators, H_2_ antagonists, anticancer agents, β factor blockers, etc. [1,2,4,9,10,11,12].

Non-steroidal anti-inflammatory medications (NSAIDs) and their metabolites have been frequently identified in different environmental matrices and are, therefore, included on the list of priority emerging pollutants [4,9,10,14,15,16]. Since only 5% of the therapeutic dose of naproxen (NPX) and its water-soluble sodium salt are fully metabolized [17,18], this aromatic emerging pollutant has been found in both wastewater as well as natural water in concentrations from 0.02 μg/L to 250 μg/L [16,19,20,21,22,23]. Specifically, in Mexico, it has been found in concentrations of 186 ng/L in surface waters [9] and 1.79 μg/L in hospital effluents [4].

These reports clearly indicate that medications and their metabolites are not fully mineralized in conventional wastewater treatment plants because most pharmaceuticals are lethal to the bacteria responsible for their oxidation. Mendez Arriaga et al. [16] and Mansouri et al. [21] reported that conventional wastewater treatment plants can only degrade between 60% and 70% of the naproxen in the influent. For this reason, it is very important to develop new chemical processes to transform recalcitrant micropollutants into other compounds that do not harm the environment [24]. Therefore, ensuring effective treatment is critical for protecting water sources for human consumption [25,26]. Most persistent organic compounds can only be oxidized through advanced oxidation processes (AOPs) which are based on the generation of reactive oxygen species (H_2_O_2_, HO^•^, O_2_^•−^, and O_3_) with the ability to mineralize recalcitrant organic compounds [3,20,21,26,27].

Several studies have demonstrated that some non-steroidal anti-inflammatory drugs (NSAIDs), such as acetaminophen, ketoprofen, and diclofenac, can be efficiently degraded and mineralized by photochemical processes using a semiconductor catalyst that upon irradiation with UV-A light undergoes a surface reaction generating electron (e_cb_^−^)–positive hole (h_vb_^+^) pairs [28,29,30,31,32,33]. These reactive species initiate several redox reactions on the surface of the catalyst and generate O_2_^•−^, HO_2_^•−^, and HO^•^ radicals which in turn oxidize any organic molecule [30,34,35,36]. In the case of naproxen, an NSAID with a more complex chemical structure than acetaminophen, ketoprofen, and diclofenac, several research papers have shown that NPX can be degraded by photochemical processes with pure TiO_2_ or sulfur-, nitrogen-, copper-, nickel-, or iron-doped TiO_2_ catalysts illuminated with UV light [14,16,37,38,39].

Mendez-Arriaga et al. [16] studied the photocatalytic degradation of naproxen with commercial TiO_2_ in a tubular reactor equipped with a xenon lamp (290 < λ < 400 nm). Their results indicated that photolysis plays an important role in the photocatalytic degradation and mineralization of naproxen. Chemical analysis of the reaction samples had shown that demethylation and decarboxylation are the initial reaction steps in the oxidation of NPX, giving some photoproducts such as 2-acetyl-6-methoxy-naphthalene and 1-(6-methoxy-2-naphthyl) ethanol. It has been reported [14,18,37,40] that the decarboxylation route also gives at least two dimerization photoproducts. Irradiation of a naproxen solution or a TiO_2_ and naproxen slurry with UV-C light increases the number of intermediate reaction products and improves the degradation efficiency [41]. Other studies have demonstrated that NPX is also successfully degraded and mineralized with TiO_2_, BiVO_4_, and metal-doped g-C_3_N_4_ catalysts illuminated with radiation with a wavelength greater than 360 nm [19,23,38,39].

Considering that naproxen (NPX) and its metabolites have been found in both fresh and residual water in high concentrations, up to micrograms per liter, the study of photochemical and photocatalytic degradation processes to remove these emerging pollutants from water bodies is important. Several photocatalytic degradation experiments were carried out with high-concentration naproxen aqueous solutions and TiO_2_ Evonik P-25, a well-known catalyst with a high photochemical activity, illuminated with UV-A light under continuous flow of molecular oxygen. Under these experimental conditions, the original reactant (NPX) is degraded to other organic molecules with a different chemical structure which in turn are fully mineralized to CO_2_ and water. This process follows a complicated reaction pathway that includes pure photochemical reactions and a series of parallel photocatalytic reactions. Other studies [16,22,40] have only identified some of the intermediate reaction products and reported partial information about the reaction mechanism of the photocatalytic degradation of naproxen. In order to elucidate the complete reaction mechanism, the most stable intermediate organic reaction products must be fully identified by several analytical techniques that include UV–Vis spectroscopy, FT-IR spectroscopy, total organic carbon analyzer, HPLC with a UV-Vis detector, HPLC with an MS detector, NMR, and direct mass spectroscopy. 

## 2. Results and Discussion

### 2.1. Naproxen Adsorption Studies

It has been previously reported that under specific pH conditions, the negatively charged carboxylate group of two other NSAID drugs, diclofenac and ketoprofen, is adsorbed on the surface of the positively charged titania surface [31,32]. Therefore, the concentrations of undissociated naproxen [HA] and its conjugated base [A^−^] as a function of pH were calculated with an Excel program using the Henderson–Hasselbalch Equation (1) which represents the logarithmic form of the mass law equation, where pK_a_ is the logarithm of the acid dissociation constant and pH is the logarithm of the reciprocal of the hydrogen ion activity.

(1) $$pH=pK_{a}+log\frac{\left[A^{-}\right]}{\left[HA\right]}$$

According to the speciation diagram (Figure S1 in the Supplementary Materials), more than 50% of the NPX in the solution has a negative charge when the pH > 4.19. Therefore, at the natural pH (6.6 ± 0.1) of the naproxen–titania slurry, all the organic molecules are negatively charged. Previous studies have indicated that the PZC of TiO_2_ P25 is located at pH = 6.35 (Figure S2) and confirmed that the surface of the photocatalyst is also negatively charged at pH > PZC [31,42,43].

Even though the experimental conditions do not favor the adsorption of the conjugated base of naproxen on the surface of the TiO_2_ P25 catalyst, the experimental values of naproxen uptake in mMol g^−1^ (q_s_) as a function of equilibrium concentration (C_eq_) in mMol L^−1^ are plotted in Figure S3. Then, the parameters of the Langmuir isotherm (Equation (2)) [31,44] for the adsorption of NPX on TiO_2_ were determined from the results of all the adsorption experiments.

(2) $$q_{s}=\frac{q_{m}K_{eq}C_{eq}}{1+K_{eq}C_{eq}}$$

Both constants, maximum uptake of NPX per gram of catalyst (q_m_) in mMol g^−1^ and Langmuir isotherm constant in (K_eq_) L mMol^−1^ of Equation (2), were calculated by the non-linear least-squares method using the Delta Graph 7 software. The numerical values are: q_m_ = 0.0362 mM NPX g^−1^ TiO_2_ and K_eq_ = 1.4707 L mMol^−1^ NPX, confirming that only a small fraction of naproxen in solution interacts with the catalyst’s surface. Mendez-Arriaga et al. [16] also carried out several NPX adsorption experiments on TiO_2_ at free pH (6.15 ± 0.15) and reported that up to 92% of the medication remained in the solution independently of the initial concentration and amount of catalyst.

In order to compare the naproxen adsorption on TiO_2_ with the adsorption of other NSAIDs, diclofenac and ketoprofen, adsorption isotherms were calculated using their previously reported Langmuir constants adsorption (Table 1) [31,32]. Both isotherms are also plotted in Figure S3. Since diclofenac and ketoprofen adsorption experiments were carried out at pH < PZC, the catalyst surface is positively charged, and organic molecules are negatively charged. These experimental conditions favor the adsorption of diclofenac and ketoprofen on the surface of the TiO_2_ catalyst. On the other hand, the naproxen adsorption experiments were carried out at pH = 6.6 ± 0.1 and both species, catalyst’s surface and organic molecules, are negatively charged. Therefore, only a small fraction of the medication is adsorbed on the catalysts.

### 2.2. Investigation of NPX Photochemical and Photocatalytic Degradation by HPLC and TOC

All heterogeneous photocatalytic reactions follow a multiple-step process that includes mass transfer of the reactants from the fluid phase to the surface of the catalysts, adsorption of those reactants onto the solid surface, transformation of the adsorbed chemical species by chemical or photochemical reactions, desorption of the reaction products, and diffusion of the products to fluid phase [33,45]. In the case of NPX, only a small fraction of the reactant in the solution is adsorbed on the surface of the TiO_2_ photocatalyst. At the beginning, by means of a predominant photochemical process, NPX is transformed into other organic molecules which in turn are adsorbed on the surface of the catalyst before being mineralized into CO_2_ and water. Then, it is very important to carry out pure photochemical degradation experiments of naproxen aqueous solutions illuminated with low-energy UV light (λ_max_ = 365 nm) and without any catalyst in order to determine, by HPLC and TOC analysis, the amount of the original reactant and the amount of carbon of all organic chemical compounds that remain in the solution.

**Figure 1:**
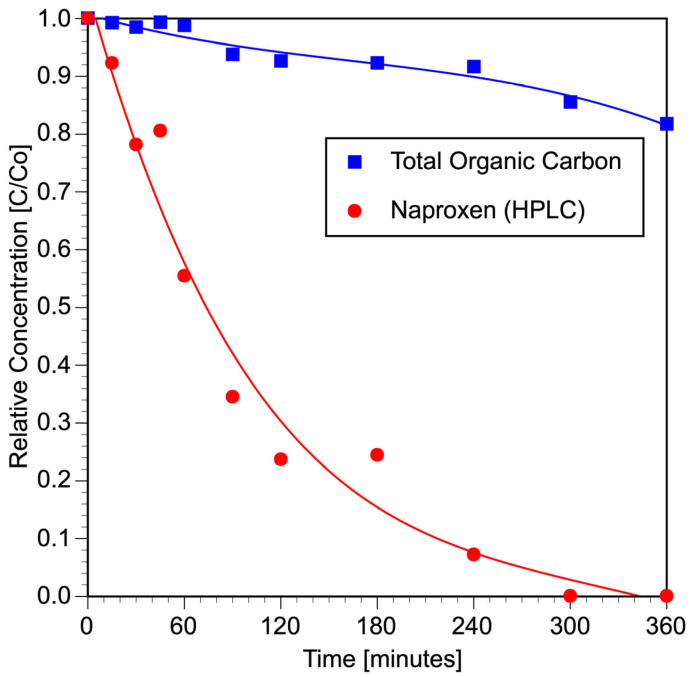
Photochemical degradation of an aqueous solution of naproxen (initial concentration = 20 ppm = 0.0793 mM, initial pH = 6.4, V = 300 mL, four UV lamps λ_max_ = 365 nm, O_2_ flow = 100 mL min^−1^, analysis of reaction samples by HPLC and TOC).

HPLC analysis of the reaction samples obtained in a photochemical oxidation experiment of a 20 ppm naproxen solution (Figure 1) clearly indicates that 100% of the reactant is fully degraded in 3 h of reaction. Under the experimental conditions of this study, only 18% of the organic compounds were fully mineralized to CO_2_ and water in six hours of reaction. Then, a simple material balance [46] indicates that 82% of naproxen was slowly transformed into other intermediate organic products (Tables S1 and S2). These results suggest that naproxen undergoes decarboxylation to yield other naphthalene derivatives with a slightly different chemical structure to the reactant. Isidori et al. [22], Méndez-Arriaga et al. [16], and Kawabata et al. [40] also reported that naproxen is completely transformed to several aromatic photoproducts by irradiation of aqueous solutions with a solar simulator equipped with a xenon lamp. However, they reported that less than 10% of the reactant is fully mineralized.

**Figure 2:**
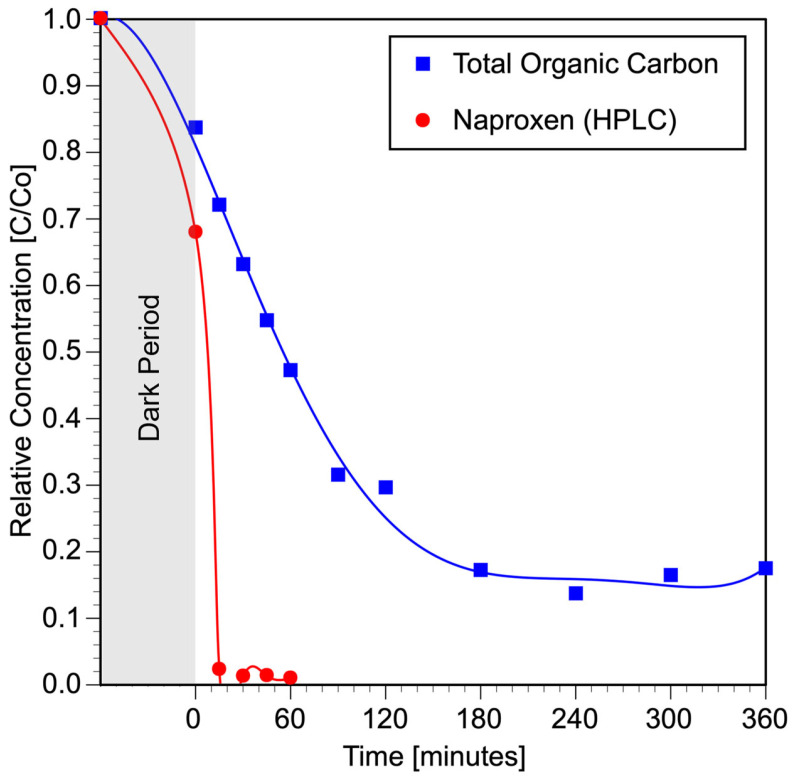
Photocatalytic degradation of an aqueous solution of naproxen (initial concentration = 20 ppm = 0.0793 mM, initial pH = 6.6, V = 300 mL, TiO_2_ = 2 g L^−1^, four UV lamps λ_max_ = 365 nm, O_2_ flow = 100 mL min^−1^, analysis of reaction samples by HPLC and TOC).

As expected, the results of the photocatalytic degradation experiments (Figure 2) show that naproxen is fully degraded during the first 15 min of reaction. Figure 2 also indicates that naproxen is easily transformed into other intermediate organic species produced during the first hour of reaction and that around 80% of all the organic molecules are mineralized to CO_2_ and water in 4 h of reaction. The remaining 20% of the original reactant is transformed into other intermediate organic products. It is very important to mention that three organic reaction products with strong absorption at 230 nm were detected at 3.77, 5.08, and 6.42 min during the HPLC analysis (Figure S4). Other researchers [16,41,47] also reported that naproxen is mineralized to CO_2_ and water in the presence of semiconductor catalysts illuminated with UV light via formation of several intermediate organic compounds.

### 2.3. UV–Vis Spectroscopy Studies

**Figure 3:**
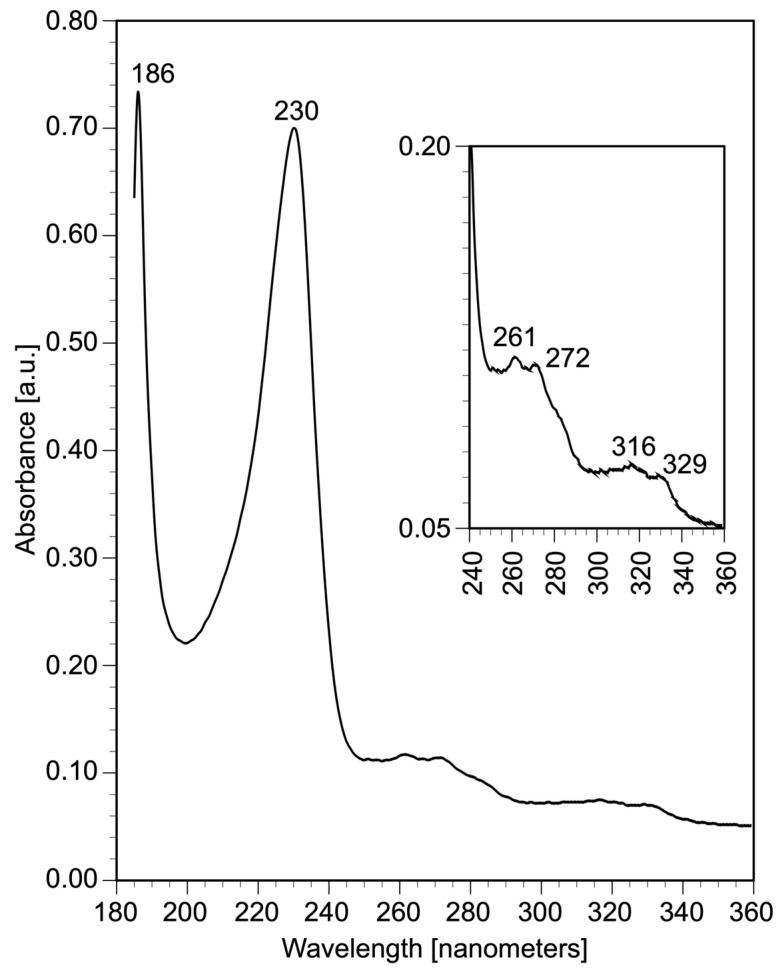
UV–Vis absorption spectrum of a naproxen aqueous solution (initial concentration = 20 ppm = 0.0793 mM, initial pH = 6.8).

The complete UV–Vis spectrum of pure NPX was obtained and analyzed (Figure 3) using information from the literature [48,49,50,51]. According to the spectrum, naproxen presents two strong absorption bands in the UV region, the most intense located at around 190 nm corresponds to the primary aromatic π–π* transition. Another band appears at 230 nm and corresponds to the secondary aromatic π–π* transition. In polynuclear aromatic hydrocarbons these bands shift to longer wavelengths relative to the bands in benzene. In addition to these two bands, the are several low-energy bands in the regions 240 to 290 and 300 to 350 nm which correspond to several forbidden *n*–π* transitions (Figure 3). These latter bands are usually associated with transitions of non-bonding electrons like the ones present in R-O-H [48].

**Figure 4:**
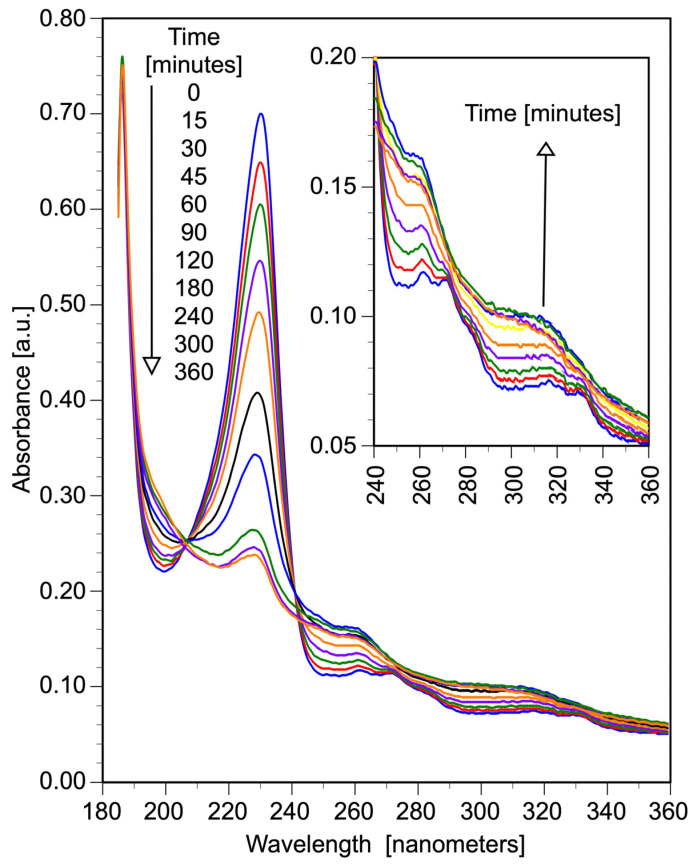
UV–Vis absorption spectra of the reaction samples of a naproxen photochemical degradation experiment (initial concentration = 20 ppm = 0.0793 mM, initial pH = 6.8, V = 300 mL, four UV lamps λ_max_ = 365 nm, O_2_ flow = 100 mL min^−1^, analysis of reaction samples by UV–Vis spectroscopy).

To gain more insight into the photochemical degradation of NPX, the full spectra from 190 nm to 800 nm of reaction samples photolyzed at different reaction times were obtained and analyzed (Figure 4). At early stages, as the photochemical reaction progresses, a hypochromic effect is observed with the band at λ = 230 nm. However, this effect is observed up to a certain point, after which all the original bands remain in the sample with a different intensity. These results indicate that NPX has been transformed into other aromatic compounds. In addition, the bathochromic and hyperchromic effects observed in the bands in the two regions from 240 to 290 and 300 to 350 nm (Figure 4) indicate the presence of an OH group in at least one of the new aromatic derivatives [28].

**Scheme 1:**
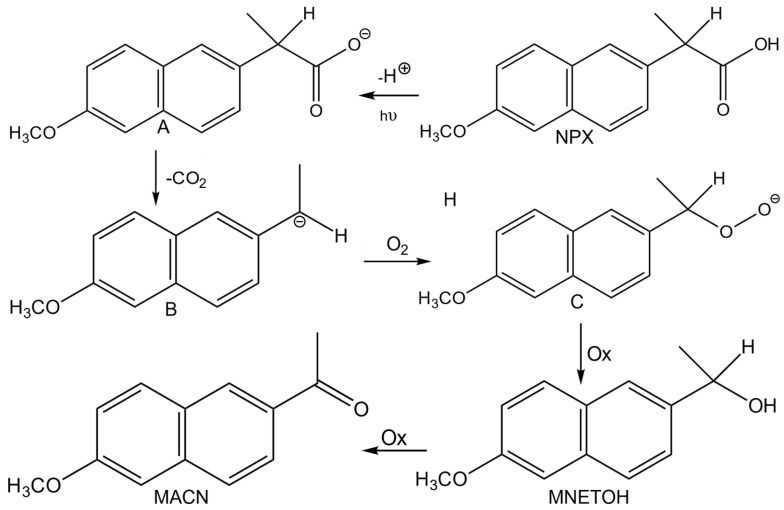
Photodegradation of NPX under illumination of UV light with λ_max_ = 365 nm [40].

There is a previous report [40] on the photochemical degradation of NPX using low-energy light (365 nm) indicating the formation of two photoproducts (Scheme 1). By means of ^1^H-NMR spectroscopy, these products were clearly identified as naphthalene derivatives, 1-(6-methoxynaphthalen-2-yl)ethan-1-one (MACN) containing an acetyl group and 1-(6-methoxynaphthalen-2-yl)ethan-1-ol (MNETOH) with an ethanol side chain. Experimental results in this investigation are in agreement with previous findings by Kawabata et al. [40]. Therefore, in the case of NPX, photolysis even with low-energy light (365 nm) leads to decarboxylation (Scheme 1) with the formation of an anion B that is eventually oxidized giving two other aromatic compounds MACN and MNETOH (Scheme 1).

**Figure 5:**
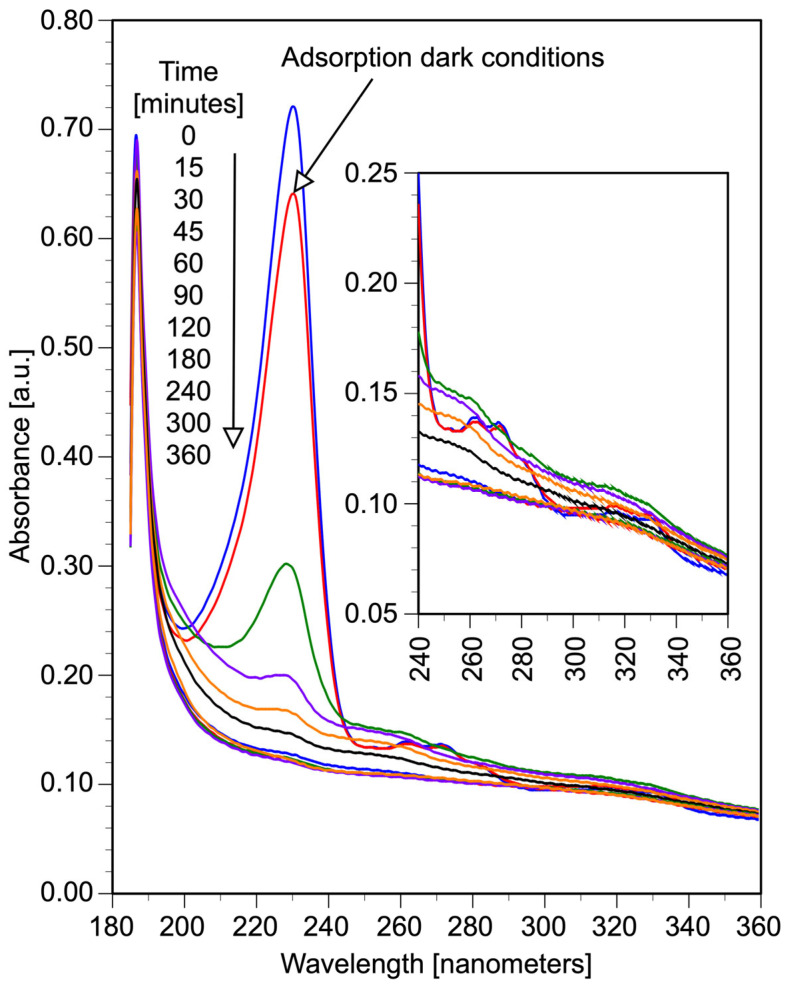
UV–Vis absorption spectra of the reaction samples of a naproxen photocatalytic degradation experiment (initial concentration = 20 ppm = 0.0793 mM, initial pH = 6.8, V = 300 mL, TiO_2_ = 2 g L^−1^, four UV lamps λ_max_ = 365 nm, O_2_ flow = 100 mL min^−1^, analysis of reaction samples by UV–Vis spectroscopy).

Before performing any photocatalytic experiments, NPX UV–Vis spectra were obtained without and with TiO_2_ catalyst (Figure 5). These results confirm that only a small amount of NPX is adsorbed on the catalyst. Therefore, at the beginning of the experiment after turning on the UV lamps, some degradation of NPX may take place in the fluid phase by photolysis as indicated in Figure 4 and Scheme 1. The intermediate organic products of the pure photochemical reactions may be adsorbed on the surface of the catalyst and react with hydroxyl radicals generated by photocatalysis.

During the photocatalytic degradation of NPX, different changes were also observed on the UV–Vis spectra of NPX. The intensity of the absorption band at 230 nm decreases very fast and reaches a minimum after 90 min of irradiation. This change indicates that NPX is transformed into other types of aromatic compounds very fast. Furthermore, the bands in the two regions (240 to 290 and 300 to 350 nm) experience bathochromic and hyperchromic effects up to 30 min of irradiation (Figure 5), indicating the insertion of hydroxyl radical in the aromatic ring. After this time, these bands also decrease in intensity. Contrastingly, the band at 190 nm does not change that much and it remains in the spectra even upon extended irradiation. Since this band is characteristic of unsaturated carboxylic acids [48], it could be concluded that NPX has been transformed into this type of compound.

### 2.4. IR Spectroscopy Studies about Photocatalytic Degradation of NPX and Intermediate Compounds

**Figure 6:**
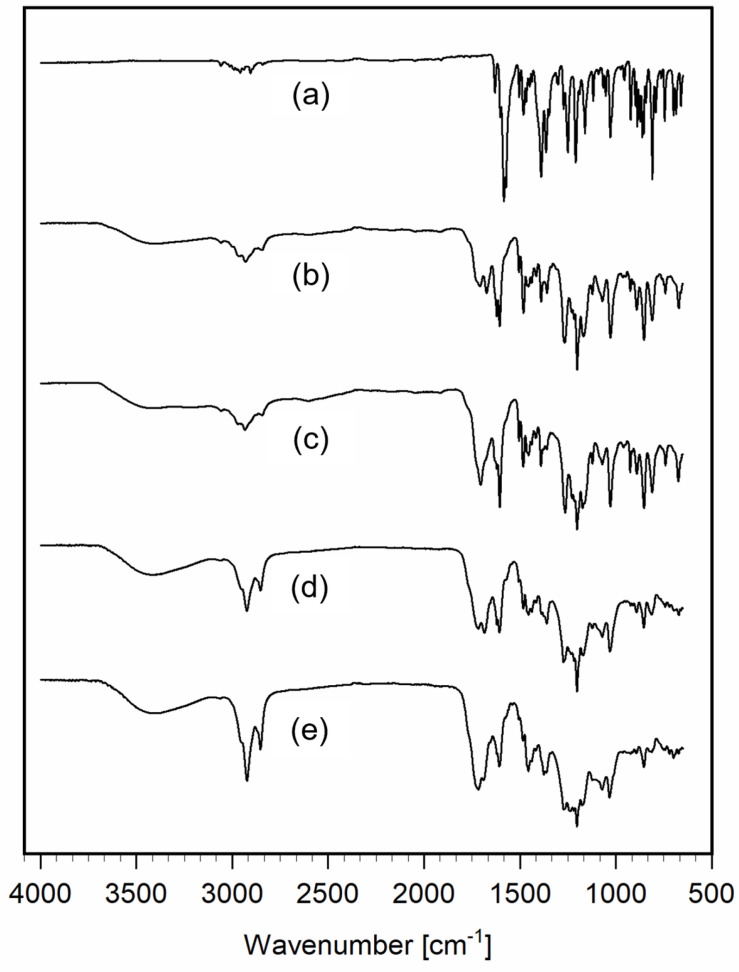
Photocatalytic oxidation of naproxen monitored by FT-IR. Samples were taken after (**a**) naproxen spectra, (**b**) 30 min of reaction, (**c**) 60 min of reaction, (**d**) 90 min of reaction, and (**e**) 180 min of reaction. (initial concentration = 100 ppm = 0.396 mM, initial pH = 6.8, V = 300 mL, TiO_2_ = 2 g L^−1^, four UV lamps λ_max_ = 365 nm, O_2_ flow = 100 mL min^−1^).

Before any experiments, an NPX sample was analyzed by FT-IR (Figure S5, Figure 6, line a) to identify its characteristic bands, taking in account its structure and functional groups present, according to the literature [48]. In this discussion, all the FT-IR vibrations bands are given in cm^−1^. Several bands (3100–3000) correspond to C-H (C sp^2^ for aromatic compounds) and other bands (3000–2837) correspond to C-H (C sp^3^ for aliphatic chain). Several bands (1633, 1600, and 1480) correspond to aromatic symmetrical and asymmetrical C=C stretching. Aromatic compounds usually present some bands in this region (1600 to 1475). Two strong bands (1583 and 1389) correspond to an asymmetric and symmetric O-C=O stretching. These vibrations usually show at lower wavelengths due to the resonance effect. An aryl alkyl ether gives two vibrations (1209 and 1029) corresponding to stretching (C-O-C). Three strong bands (862, 855, and 809) correspond to C=C-H out-of-plane bending of aromatic compounds.

In order to obtain reaction samples for FT-IR analysis, a volume of an NPX solution was irradiated for a certain amount of time. Then, it was extracted with an organic solvent and the organic phase was dried and the solvent removed to give a solid mixture. Several changes are observed between the pure NPX sample (Figure S5, Figure 6, line a) and the sample of the photocatalytic reaction mixture after 30 min of irradiation (Figure S5, Figure 6, line b). A characteristic O-H stretching band from 3100 to 3650 is present. This O-H band is observed in aromatic alcohols around 3600, however, this band could also be due to the presence of carboxylic acids. Strong and well-resolved bands are observed in the range 2800 to 3070, indicating the presence of compounds with carbons with both sp^2^ and sp^3^ hybridization. Therefore, part of the NPX structure has broken into smaller aromatic and aliphatic compounds. Two strong and broad bands are observed at 1704 and 1674, indicating the presence of several compounds containing a C=O (ketones and/or carboxylic acids). Further evidence of the presence of carboxylic acids are the band at 1266 due to C-O stretching and the band at 927 due to O-H bending. The characteristic C=C aromatic stretching bands observed at 1620 and 1606 increased in intensity since the NPX structure has been cleaved into smaller aromatics. Further evidence of the presence of aromatic structures are vibrations in two more regions. Weak overtone combination bands observed in the region 2000 to 1667 and several strong bands observed (891, 853, and 810) are due to C=C-H bending out of plane. In the sample, an aryl alkyl ether structure still remains since the two vibrations (1202 and 1029) corresponding to stretching (C-O-C) are clearly observed.

In the IR spectrum of the photocatalytic sample after 90 min of irradiation (Figure S5, Figure 6, line d) a strong and broad O-H stretching band is clearly observed (3650–2400) indicating the presence of several hydroxylated compounds (carboxylic acids, alkyl and/or aryl phenols). Further evidence of this are the several bands (1300 to 1100) characteristic of various types of C-O stretching. Furthermore, two bands (1716 and 1685) indicate the presence of several groups containing C=O (ketones and/or carboxylic acids). The bands corresponding to C-H (C sp^3^) have become stronger, indicating the formation of low-molecular-weight compounds. In addition, the bands around 1600 characteristic of C=C-H bending of aromatics have become smaller.

The IR spectrum of the photocatalytic reaction mixture after three hours of reaction (Figure S5, Figure 6, line e) is very similar to the previous one with only some variations in the intensity of the signals. The two broad signals (1716 and 1710) due C=O are more intense. There are no overtones characteristic of aromatics (2000 to 1667). The band around 1600 is due to C=C stretching in alkenes since aromatic compounds usually present several bands in this region. There are more bands due to different types of C-O (1170 to 1300) vibrations. Based on this analysis, it could be concluded that NPX has been transformed into saturated and unsaturated low-molecular-weight acids. 

**Figure 7:**
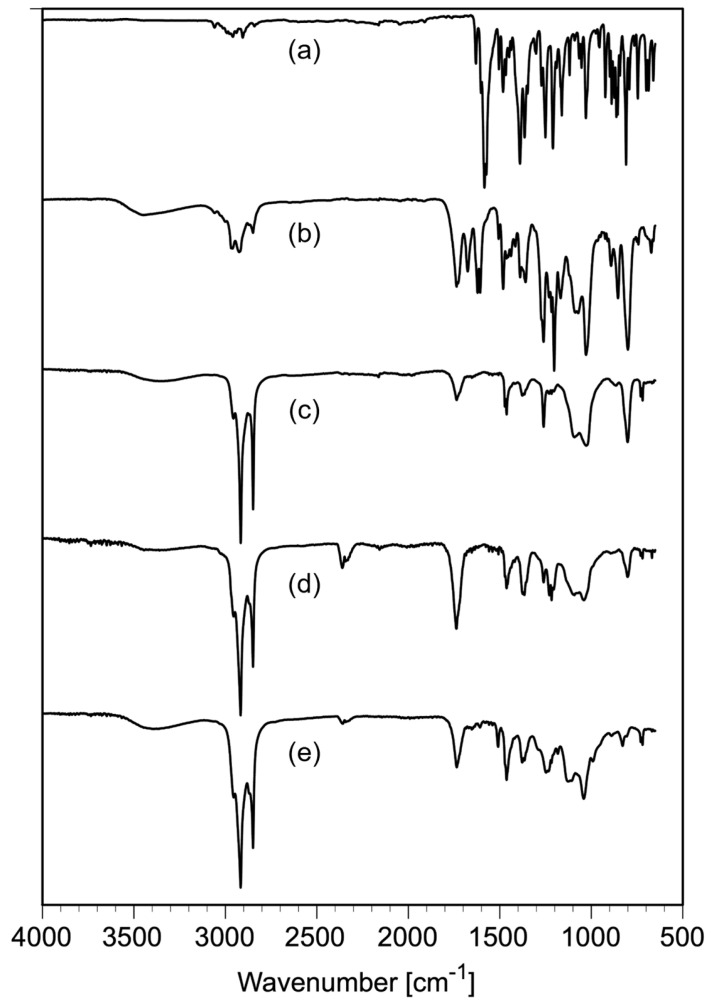
FT-IR spectra of the TLC fractions of the organic extract of a sample taken after 60 min of reaction: (**a**) naproxen spectrum, (**b**) most polar fraction 1, (**c**) fraction 2, (**d**) fraction 3, and (**e**) least polar fraction 4 (initial concentration = 100 ppm = 0.396 mM, initial pH = 6.8, V = 300 mL, TiO_2_ = 2 g L^−1^, four UV lamps λ_max_ = 365 nm, O_2_ flow = 100 mL min^−1^).

Since the reaction mixture after 60 min of irradiation gave the largest yield of intermediate compounds (Figure S5, Figure 6, line c), this particular sample was separated into four fractions by thin layer chromatography (TLC) using a mixture of hexane and ethyl acetate as a moving phase. Four different crystalline fractions were obtained and analyzed. The IR spectra of these fractions (Figure S6, Figure 7, lines b, c, and d) were analyzed in detail.

In the IR spectrum of the first fraction (Figure S6, Figure 7, line b) there is a broad band (3650 to 3700) characteristic of O-H stretching of hydroxylated species (alkyl and/or aryl carboxylic acids and/or alcohols). Several bands (3060 to 2800) indicate the presence of various compounds with C-H (C sp^2^ and sp^3^). Three strong bands (1725, 1720, and 1680) correspond to C=O stretching (ketones and/or carboxylic acids). Two bands located around 1600 correspond to C=C stretching of aromatics. The band at 1660 corresponds to C-O of a carboxylic acid while the other two bands in this region (1202 and 1028) correspond to vibrations of alkyl aryl ether C-O-C. The vibration around 799 corresponds to an O-H bending. There are several bands (1000–1100) corresponding to the C-O vibration of alkyl or aryl alcohols. Therefore, this fraction contains a mixture of compounds (alkyl and aryl) acids and alcohols.

The other three fractions (Figure S6, Figure 7, lines c, d, and e) have a very similar spectrum. Only the FT-IR spectra of line d (Figure 7) is analyzed in detail. This sample presents a broad band (3650–2800) corresponding to O-H stretching. Strong bands located between 3000 and 2700 correspond to C-H stretching (C sp^3^). Very weak bands (3000 to 3100) corresponding to C-H stretching (C-H sp^2^) and a broad band (around 1730) corresponding to a C=O stretching of carboxylic acids are also evident. The strong band at 1450 corresponds to C-H bending of CH_2_ groups. There are several well-defined C-O stretching bands (around 1200) and some strong C=C-H bending bands (1100 to 700). In these latter TLC fractions, there are mixtures of saturated and unsaturated low-molecular-weight carboxylic acids.

### 2.5. Studies on pH on NPX Photocatalytic Degradation

**Figure 8:**
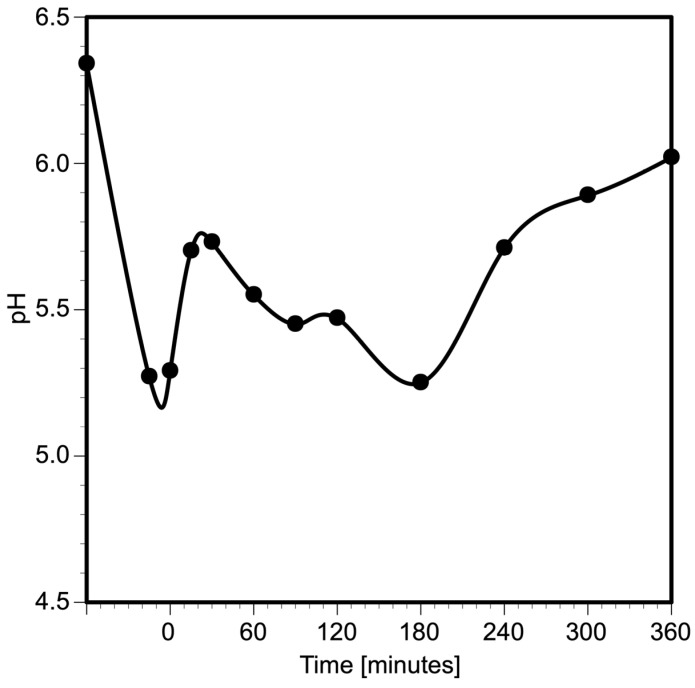
Variation of pH during the photocatalytic degradation of a naproxen aqueous solution. (Initial concentration = 20 ppm = 0.0793 mM, initial pH = 6.4, V = 300 mL, four UV lamps λ_max_ = 365 nm, O_2_ flow = 100 mL min^−1^).

FT-IR analysis of the reaction samples of NPX photocatalytic degradation gave strong evidence of its mineralization via formation of carboxylic acids. To obtain further evidence of this reaction pathway, the pH was measured at different times of irradiation on NPX photocatalytic degradation mixtures (Figure 8). The initial pH of an NPX solution of 20 ppm NPX solution was 6.34. Since NPX has a low pK_a_ value of 4.19 [52], this compound will be present in an ionic state at the beginning of the experiment (Figure S1). Upon the addition of the catalyst, the sample changes to a carboxylic acid and the slurry pH changes to a lower value (5.3). During the first thirty minutes of irradiation, an increment in pH value up to 5.8 is observed. This change indicates that NPX is transformed into less acidic compounds. It is well documented that upon irradiation with low-energy UV light (350 nm) NPX is decarboxylated and transformed into other aromatic compounds [40]. After this time, the pH slowly decreases and reaches its lowest point (5.2) at 180 min. These experimental results indicate that intermediate organic compounds generated at the beginning are being transformed into other types of compounds. This later pH is quite close to the pH obtained for aromatic and aliphatic acids [53]. In the range of 180–300 min of irradiation, the pH slowly increases up to 5.9, indicating that these latter carboxylic acids are also degraded into more basic compounds.

### 2.6. ^1^H NMR Studies on NPX Photocatalytic Degradation

**Figure 9:**
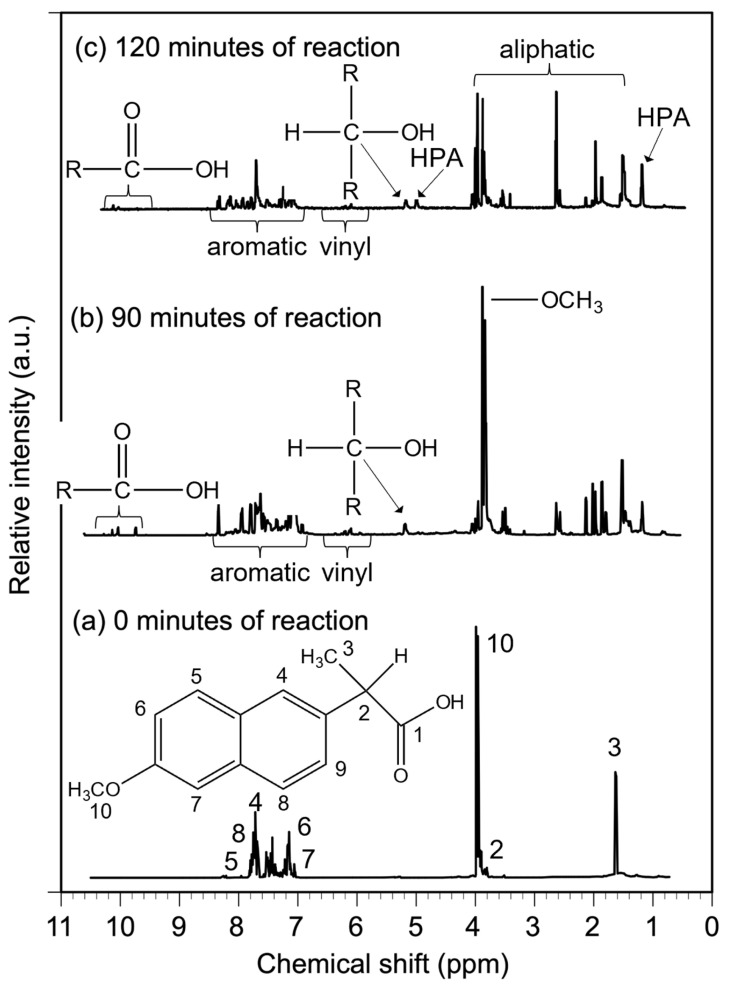
Photocatalytic oxidation of naproxen monitored by ^1^H-NMR. Samples were taken at 0, 90, and 120 min of reaction (initial concentration = 200 ppm = 0.793 mM, initial pH = 6.8, V = 300 mL, TiO_2_ = 2 g L^−1^, four UV lamps λ_max_ = 365 nm, O_2_ flow = 100 mL min^−1^).

To investigate the chemical changes involved in the photocatalytic degradation of NPX, samples of the reaction mixture were taken at different times and analyzed by ^1^H NMR. Several aromatic intermediate reaction products are generated during the photoconversion of NPX, and they have been clearly identified by ^1^H NMR [18,40]. The summary of the signals observed for each compound is given in Table 2. The ^1^H NMR spectra of NPX and three samples of a photocatalytic degradation experiment are presented in Figure 9 and Figure S7. Several general observations could be obtained from these spectra. Naphthalene hydrogens give signals in the range 7 to 9 ppm. Aliphatic hydrogens give signals in the range 1 to 3 ppm. A hydrogen on a carbon next to a C=O is deshielded and gives signals close to 4 ppm. A hydrogen in an alcohol H-C-OH gives signals around 5 ppm. Hydrogens attached to oxygen of an OCH_3_ group are highly deshielded and give a singlet around 3.9 ppm. Vinyl protons are usually highly deshielded due to the anisotropy of the double bond, giving signals in the range 5.5 to 7 ppm.

First, the ^1^H NMR of the starting material was obtained and analyzed, and all the signals were assigned based on a previous report [40]. In the spectra of starting material and reaction mixtures, the position of each hydrogen is given in ppm. To determine the types of organic compounds present in reaction mixtures, all the ^1^H NMR signals were assigned based on the literature [48].

The molecular structure and ^1^H NMR of NPX is given in Figure 9. The hydrogen on the carboxylic acid did not give any signal. In general, this hydrogen gives a very weak signal, if any, since it easily exchanges with hydrogens in solvent. In the structure of NPX there are several aliphatic protons. A doublet at 1.59 ppm corresponds to hydrogens on carbon **3**, a quadruplet at 3.87 ppm corresponds to hydrogen on carbon **2**, and a highly deshielded singlet signal at 3.91 ppm corresponds to hydrogens on carbon **10** of an OCH_3_ group. All the aromatic protons gave signals in the corresponding region (7 to 9 ppm). A doublet at 7.10 ppm corresponds to hydrogen on carbon **7**, a doublet of doublets at 7.14 ppm corresponds to hydrogen **6**, a doublet of doublets at 7.41 corresponds to hydrogen on carbon **9**, a singlet at 7.68 ppm corresponds to hydrogen on carbon **4**, a doublet at 7.69 ppm corresponds to hydrogen on carbon **8**, and a doublet at 7.71 corresponds to hydrogen on carbon **5**.

The reaction mixture photolyzed for 90 min still presents all the signals corresponding to NPX (Figure S7, Figure 9, line b). Several changes are observed in this mixture. Around 3.8 ppm, there are several highly deshielded signals indicating the presence of several compounds containing an O-CH_3_ substituent. There are several signals in the range from 1 to 3 ppm corresponding to some hydrogens in aliphatic carbons indicating that in a certain number of molecules, the two aliphatic chains in NPX have been separated from the naphthalene ring. Further evidence of this is the fact that the aromatic region (7 to 9 ppm) has become quite complex, indicating the presence of several different aromatic compounds. At 9.75 ppm, a signal corresponding to an aldehyde is observed. In the range from 10 to 11 ppm several signals, corresponding to carboxylic acids, are observed. There are some signals in the range from 5.5 to 7.0 ppm indicating the presence of several vinyl hydrogens. However, hydrogens on carbons containing OH like alcohols are highly deshielded and give signals around 5 ppm. Therefore, some aliphatic chains have been oxidized to low-molecular-weight acids and/or alcohols.

The samples photolyzed for 120 and 180 min gave very similar spectra. Only the sample for 120 min is analyzed (Figure S7, Figure 9, line c). All the signals in the range (7 to 9 ppm) are rather small, indicating most of the aromatic structure has been degraded to smaller compounds. All the signals associated with starting NPX are very small. Some signals in the range from 10 to 11 ppm, associated with carboxylic acids, are still present in the sample. Strong signals in the sample are observed in the range of aliphatic hydrogens (1 to 3.5 ppm). In addition, several signals are also observed in the range (5.5 to 7.0 ppm) corresponding to vinyl hydrogens. Furthermore, several signals associated with hydroxyl propionic acid (HPA) are present in this mixture 1.3 and 4.7 ppm. Signals around 5.0 are associated with hydrogens in alcohols (H-C-OH). These ^1^H-NMR results clearly indicate that the NPX structure (aliphatic and aromatic) has been degraded into low-molecular-weight saturated and unsaturated acids and/or alcohols.

### 2.7. Studies on Photochemical and Photocatalytic Degradation of NPX Using HPLC, HPLC-MS, and Direct MS

**Figure 10:**
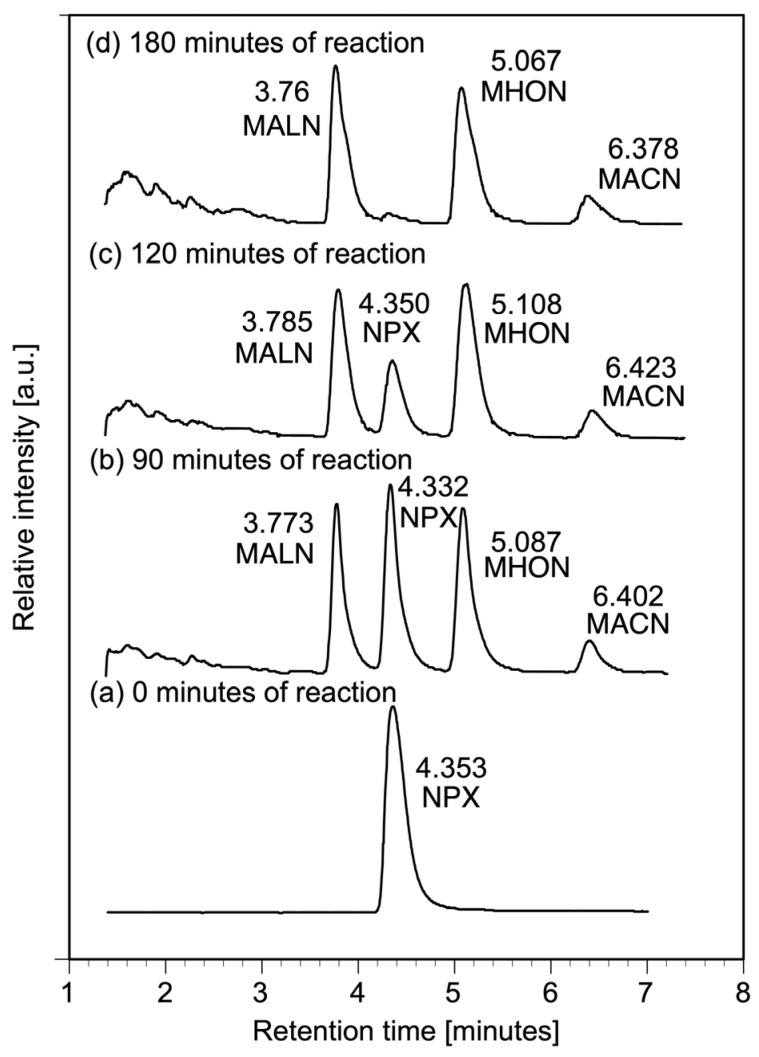
Photocatalytic oxidation of naproxen monitored by HPLC with UV–Vis photo array detector. Samples were taken at 0, 90, 120, and 180 min of reaction (initial concentration = 200 ppm = 0.793 mM, initial pH = 6.8, V = 300 mL, TiO_2_ = 2 g L^−1^, four UV lamps λ_max_ = 365 nm, O_2_ flow = 100 mL min^−1^, dilution factor for HPLC analysis = 1:10).

A volume (250 mL) of an aqueous solution of NPX (200 ppm) containing TiO_2_ (2 g L^−1^) was irradiated for different times (90, 120, and 180 min). After this time, the solution was filtered to remove any solid and it was diluted (1:10) with deionized water. Then, it was analyzed in an HPLC instrument to identify intermediate reaction products. The chromatograms of an aqueous solution of NPX and three reaction samples are shown in Figure 10. Pure NPX gives a clean chromatogram with a broad peak at 4.35 min (Figure 10, line a). After 90 min of irradiation several changes are observed. There are small amounts of different highly polar low-molecular-weight compounds in the range from 1.4 to 2.7 min. In addition to an NPX signal at 4.35 min, three other compounds are detected in the reaction mixture (Figure 10, line b): 6-methoxy-2-naphthaldehyde (MALN), 6-methoxynaphthalen-2-ol (MHON), and 1-(6-methoxynaphthalen-2-yl)ethan-1-one (MACN). Upon extended irradiation (Figure 10, lines c and d), the amount of all of these intermediates decreases. Results of the HPLC analysis also indicate that the amount of highly polar low-molecular-weight compounds increases with reaction time.

**Figure 11:**
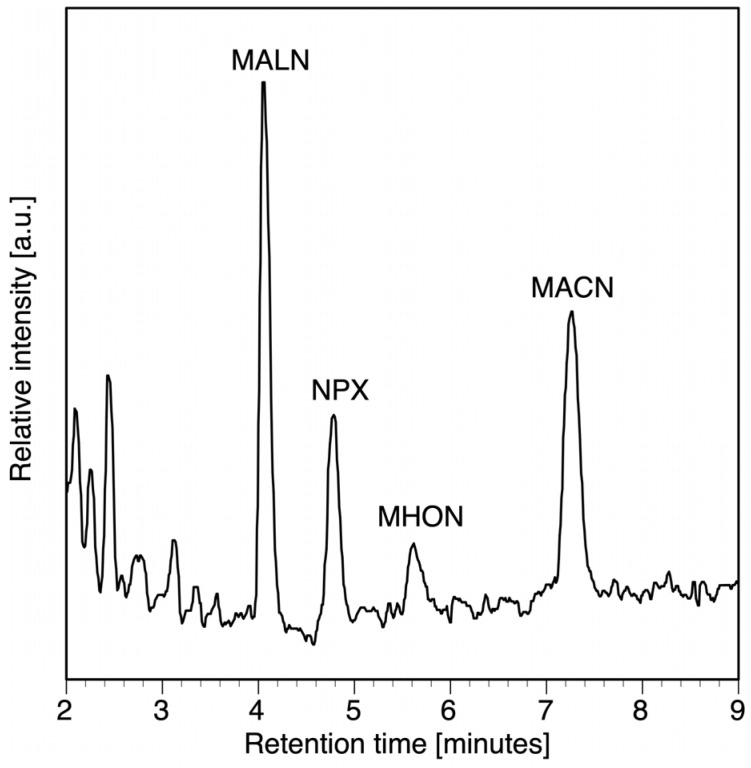
Chromatographic analysis with an HPLC-MS apparatus of a reaction sample taken after 90 min of reaction of a naproxen photocatalytic degradation experiment (initial concentration = 200 ppm = 0.793 mM, initial pH = 6.8, V = 300 mL, TiO_2_ = 2 g L^−1^, four UV lamps λ_max_ = 365 nm, O_2_ flow = 100 mL min^−1^, dilution factor for HPLC analysis = 1:10).

**Figure 12:**
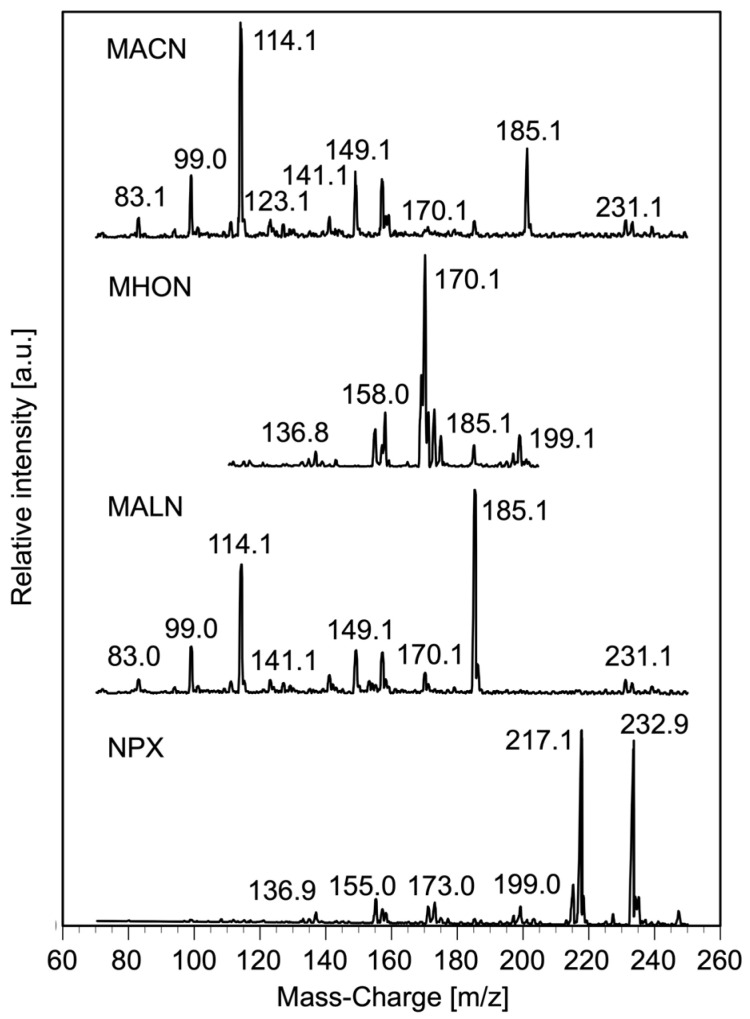
Mass spectra of naproxen and three of the intermediate organic products separated by HPLC in a reaction sample taken after 90 min of reaction of a naproxen photocatalytic degradation experiment. (Initial concentration = 200 ppm = 0.793 mM, initial pH = 6.8, V = 300 mL, TiO_2_ = 2 g L^−1^, four UV lamps λ_max_ = 365 nm, O_2_ flow = 100 mL min^−1^, dilution factor for HPLC analysis = 1:10).

The reaction mixture obtained for NPX photocatalytic degradation for 90 min was also analyzed in HPLC-MS equipment to obtain a complete identification of intermediate compounds. The chromatogram of this reaction sample is shown in Figure 11. Each intermediate gave the corresponding M + 1 value expected for the proposed structure: 6-methoxy-2-naphthaldehyde (MALN), 6-methoxynaphthalen-2-ol (MHON), and 1-(6-methoxynaphthalen-2-yl)ethan-1-one (MACN). Several fragments were also identified in each spectrum during the MS analysis. The results of this analysis are given in Table 3 and Figure 12.

**Figure 13:**
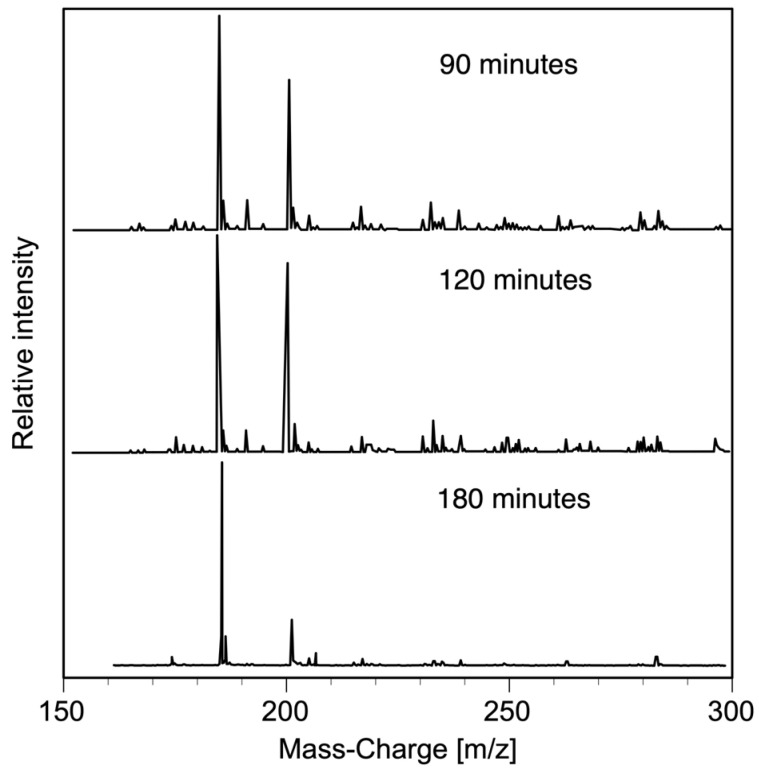
Mass spectra of the organic extract of samples taken at different reaction times during a naproxen photocatalytic degradation experiment. (Initial concentration = 100 ppm = 0.396 mM, initial pH = 6.8, V = 300 mL, TiO_2_ = 2 g L^−1^, four UV lamps λ_max_ = 365 nm, O_2_ flow = 100 mL min^−1^).

In order to gain an insight on the intermediates generated in the photocatalytic degradation of NPX at different reaction times, three solutions were prepared and irradiated as previously described (90 min, 120 min, and 180 min). Each mixture was extracted with ethyl acetate to give an organic solution. This latter solution was dried, and the solvent was removed in a roto-evaporator to give an oily sample. The results of the chemical analysis of each sample in an MS instrument are shown in Figure 13. Each compound in the reaction samples was identified for the expected MS value according to its structure. The results of MS analysis are given in Table 4. 2-(6-hydroxynaphthalen-2-yl) propanoic acid (HONPX), MACN, MALN, and MHON were detected in the three samples, but tetrahydroxy naphthalene (THON) was only detected in the reaction mixtures at longer times (120 and 180 min).

### 2.8. Intermediates and Total Degradation Mechanisms for NPX

Several authors have reported the photochemical degradation of naproxen (NPX) as either free acid or sodium salt with different light sources [14,22,30,40,54]. Performing the naproxen degradation experiments under the illumination of low-energy UV light (365 nm), only two naphthalene derivatives were detected by HPLC analysis and fully identified by ^1^H-NMR, namely 1-(6-methoxynaphthalen-2-yl)ethan-1-one (MACN) and 1-(6-methoxynaphthalen-2-yl)ethan-1-ol (MNETOH) [40]. It is very important to mention that only these two naphthalene derivatives resulting from decarboxylation and subsequent oxidation were obtained during the photochemical NPX degradation without formation of any polymeric material. Based on these results, Kawabata et al. [40] proposed a degradation mechanism involving the formation of an anion that easily oxidizes (Scheme 1). In other photochemical investigations, naproxen aqueous solutions were irradiated with either a xenon lamp or a low-pressure Hg lamp with a wide spectral output from 200 nm to 650 nm. Under those reaction conditions, several dimers are formed indicating the formation of diverse radicals [16,18,22]. For example, the photolysis of phenyl acetic acid, a compound structurally related to NPX, with high-energy UV light (210 to 280 nm) was investigated by Meiggs and Miller [55] using a flash photolysis apparatus. Their experimental results clearly indicated that phenyl acetic acid was cleaved, giving diverse radicals like benzyl radicals that promote the formation of several recalcitrant dimeric compounds. There are several reports about NPX photocatalytic degradation under different experimental conditions [14,16,19,37,39,41]. In some of these studies, several intermediates have been identified using HPLC-MS, MS, and NMR spectroscopy. Due to experimental conditions, in most of these investigations several recalcitrant dimeric compounds have been obtained along with only partial NPX degradation. Therefore, in the present investigation, NPX photocatalytic degradation experiments were carried out with TiO_2_ (Evonik P25) illuminated with low-energy UV light (λ_max_ = 365 nm) under continuous flow of molecular oxygen to avoid the formation of any recalcitrant dimeric compound. To obtain a better understanding on the actual intermediates and degradation mechanisms involved in this particular process, the degradation was investigated using a combination of analytical techniques. 

TOC studies indicated that NPX photolysis with low-energy lamps (365 nm) only leads to the formation of other aromatic compounds with a small percentage of mineralization (20%) after several hours of irradiation. Experimental results of the photocatalytic degradation of NPX with TiO_2_ (Evonik P25) indicated that the original reactant is mineralized via formation of other more recalcitrant compounds which are eventually mineralized to CO_2_ and water. In most of the experiments, at least 80 % of mineralization is achieved in six hours of reaction. 

UV–Vis spectra of the reaction samples of NPX photolysis show several bands associated with the formation and degradation of hydroxylated aromatic compounds. Similar observations were obtained following NPX photocatalytic degradation by UV–Vis spectroscopy. In this case, the associated bands with the hydroxylated aromatic compounds disappear at shorter reaction times. 

FT-IR and ^1^H NMR studies on NPX photocatalytic degradation demonstrated that this reactant is transformed into several aromatic compounds containing C=O groups (aldehyde, ketone, or carboxylic acid) and HO groups. Eventually, all compounds in the reaction slurry are degraded into low-molecular-weight saturated and unsaturated acids. 

In previous investigations on NPX photochemical and photocatalytic degradation [14,16,18,19,37,39,40,41,54], several intermediates have been detected using HPLC-MS, direct MS, or NMR analysis. A summary of all intermediates detected in this and other previous studies is reported in Table 5. Using literature information on the degradation of NPX and other structurally related aromatic compounds, several reaction mechanisms involved in NPX photocatalytic degradation are discussed.

**Scheme 2:**
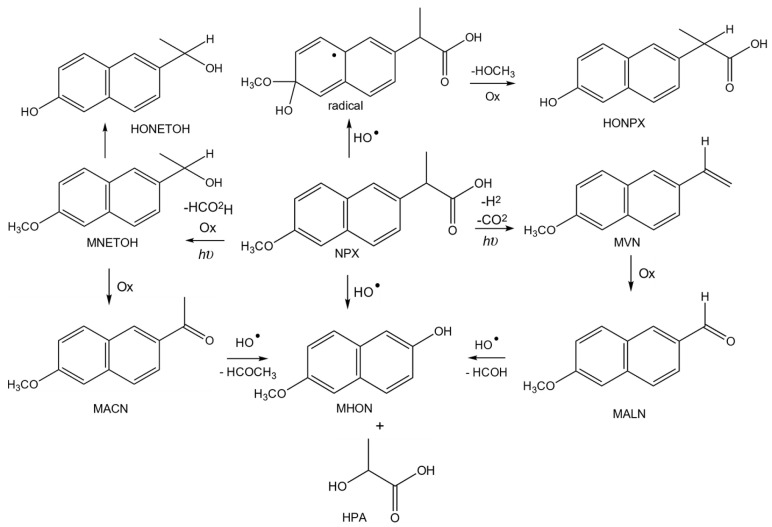
Simultaneous photocatalytic and photochemical degradation of NPX in early stages.

Based on Kawabata et al.’s [40] investigations, it is clear that at the beginning of the NPX degradation, the reaction takes place by a combination of photochemical and photocatalytic processes following a series–parallel mechanism (Scheme 2). It is well known that an aromatic structure is very prone to react with a hydroxyl radical to generate an intermediate radical [R] which eventually generates a hydroxylated naphthalene intermediate. Photolysis with low-energy radiation induces decarboxylation [22,40] and subsequent benzylic side chain oxidation leads to the formation of an alcohol, aldehyde, and ketone. Under photocatalytic conditions, these intermediates oxidize, leading to the formation of a common hydroxylated naphthalene derivative (MHON). This latter intermediate must be rather recalcitrant since its mineralization requires several oxidation steps. Further evidence of this reaction pathway is the fact that this intermediate was detected in reaction samples taken at different irradiation times.

**Scheme 3:**
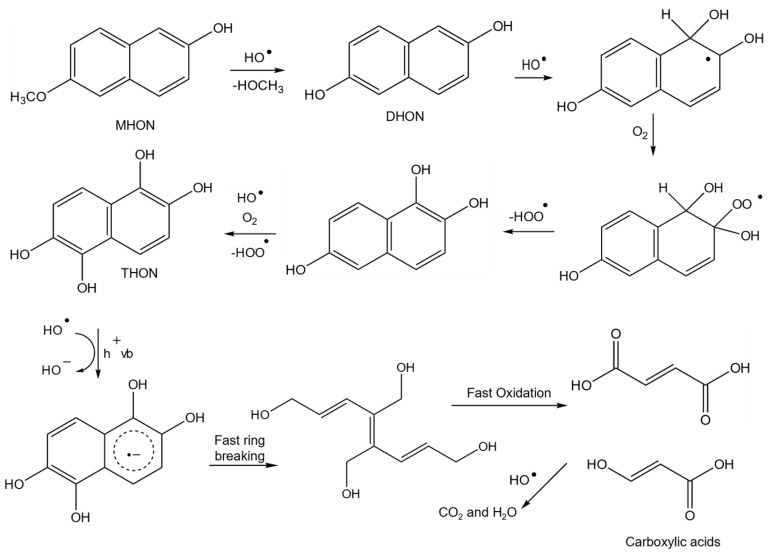
Photocatalytic degradation of NPX in latter stages.

Several studies on NPX photocatalysis indicate that the primary oxidation processes involve the participation of hydroxyl radicals (HO^•^) in the solution. According to Theurich et al. [57], positions 1, 2 and 4, 5 in a naphthalene molecule are easily oxidized under photocatalytic conditions. For example, 1-naphthol or 2-naphthol are transformed into 1,2-naphthodiol. Therefore, upon subsequent oxidation reactions mono-, di-, tri-, and tetrahydroxy naphthalene derivatives are generated in solution (Scheme 3). It has been reported in the literature that these aromatic compounds are adsorbed very strongly onto the TiO_2_ surface and as a consequence they could be oxidized by an electron transfer mechanism involving the holes (h^+^) of the catalyst and the naphthalene ring [58,59]. By this latter mechanism, the aromatic ring structure breaks very easily and generates low-molecular-weight carboxylic acids.

## 3. Materials and Methods

### 3.1. Materials

High-purity naproxen sodium salt (S)-6-methoxy-α-methyl-2-naphthalene acetic acid sodium salt, chemical formula: C_14_H_13_NaO_3_ and CAS number: 26159-34-2, was purchased from Merck. Acetonitrile and methanol, chromatographic grade, were purchase from J.T. Baker. High-purity glacial acetic acid, ethyl acetate, and anhydrous sodium sulfate were also purchased from J.T. Baker. Deionized water, filtered through 0.45 µm H.A. cellulose acetate membranes (Millipore Corp. Bedford, MA, USA), was used throughout all experiments. Titanium dioxide (Evonik P25), a known mixture of 80% anatase and 20% rutile with a surface area of about 50 m^2^g^−1^ and average particle diameter between 21 and 30 nm with E_g_ ≈ 3.12 [34,35,36,60,61,62,63,64], was used as a catalyst for all the photodegradation experiments. 

### 3.2. Photocatalytic Reactor System

Photocatalytic and photochemical degradation experiments of several organic compounds were carried out in a homemade reactor system that has already been described [46,65]. It is equipped with a 400 mL Pyrex glass beaker and four long-wave UV-A light lamps (15 W nominal power, Vilbert-Loumat). The emission spectra extend from 300 to 600 nm with three UV light maximum peaks at 352, 365, and 405 nm with three additional peaks in the visible region located at 436, 546, and 579 nm. This reactor system is also equipped with a gas feed line to bubble either an inert gas, air, or pure oxygen through the reaction mixture.

### 3.3. Adsorption Experiments

To obtain information about the actual chemical structure of naproxen in solution and its adsorption onto the surface of TiO_2_ (Evonik P25), the speciation diagram of the dissociation reaction (3) of this medication as a function of pH was determined with the mass law Equation (4) using a pK_a_ value of 4.19 [1,52].

(3) $$HA⇌H^{+}+A^{-}$$

(4) $$k_{a}=\frac{\left[H^{+}\right]\left[A^{-}\right]}{\left[\mathrm{HA}\right]}$$

where HA is the undissociated acid, A^−^ is the conjugated base, H^+^ is the hydrogen ion, and k_a_ is the acid dissociation constant. A full description of the mathematical method to determine the fraction of the undissociated medication and its conjugated base has been previously reported [31]. Since the electrical charge of the adsorbent also plays an important role in the adsorption process, the point zero charge (PZC) of TiO_2_ (Evonik P25) has been previously determined by the acid–base titration method [31].

The first set of adsorption equilibrium experiments was carried out at room temperature (22 °C ± 3 °C) under dark conditions. In each experiment, 250 mL of an aqueous solution of naproxen (NPX) with specific concentrations ranging from 10 to 100 ppm (0.039–0.396 mM) was placed in an Erlenmeyer Pyrex flask and mixed with 0.5 g of titania catalyst. Under these experimental conditions, the pH of the naproxen–TiO_2_ slurry was around 6.6. Similarly to previous work, the slurry was kept under vigorous agitation for 90 min [66] or more to allow adsorption equilibrium. Samples were taken at 15, 30, 60, and 90 min and filtered through a 0.22 μm G.V. cellulose acetate membrane (Millipore Corp., Bedford, MA, USA). The concentration of naproxen in the clear solution was determined by TOC and UV–Vis spectroscopy.

Additionally, other adsorption experiments were carried out with double and half the catalyst load, i.e., 4 g L^−1^ and 1 g L^−1^ and 100 mL of naproxen solutions with concentrations of 25, 50, and 100 ppm (0.098, 0.195, and 0.396 mM). Samples were also taken at 15, 30, 60, and 90 min, filtered, and analyzed by TOC.

### 3.4. Photochemical and Photocatalytic Degradation Experiments

To determine the effect of UV radiation on the transformation of the medication (NPX) molecule, a 20 ppm (0.0793 mM) aqueous solution of naproxen sodium salt was exposed to radiation from four UV-A light lamps. For this purpose, 300 mL of NPX solution was placed in the Pyrex beaker of the photocatalytic reactor and illuminated with four UV lamps under continuous flow of 100 mL min^−1^ of oxygen for six hours. Samples of 5 mL were taken at different reaction times and analyzed by UV–Vis spectroscopy, high-performance liquid chromatography (HPLC), and TOC. These photochemical degradation experiments were conducted in triplicate.

In most of the NPX photocatalytic degradation experiments, 300 mL of aqueous solution with a specific initial concentration of 20 ppm (0.0793 mM) or higher was placed inside the Pyrex beaker and perfectly mixed with 0.5 g of TiO_2_ (Evonik P25) under dark conditions and a continuous oxygen flow of 100 mL min^−1^ for at least 60 min. Previous studies of the photocatalytic degradation of paraquat, methyl parathion, acetaminophen, and other aromatic compounds [32,65,67] have indicated that the fastest mineralization reaction rate for these aromatic compounds is achieved with a catalyst load of 2 g L^−1^ and a gas flow of 100 mL min^−1^. Once the adsorption equilibrium time has passed, the photocatalytic reaction begins by turning on the UV light lamps to induce the formation of the electron–hole pair and to initiate redox reactions. Samples were taken at 15, 30, 45, 60, 90, 120, 180, 240, 300, and 360 min of reaction. Before analysis, all reaction samples were filtered through a Millipore 0.2 μm G.V. cellulose acetate membrane. The samples were also analyzed by UV–Vis, TOC, and HPLC [32]. These photocatalytic degradation experiments were also conducted in triplicate. Since high-concentration naproxen solutions exhibit a strong molar absorptivity, samples of the experiments carried out with NPX solutions with concentrations above 20 ppm must be diluted (1:10) before being analyzed by UV–Vis and HPLC.

### 3.5. Product Studies

Other NPX photocatalytic degradation experiments with 200 ppm (0.793 mM) solution were carried out in the same manner for specific reaction times (90, 120, and 180 min) to identify some of the intermediate organic reaction products. In this case, the photochemical and photocatalytic reactions are quenched by turning off the UV lamps. A small fraction of the reaction mixture was also filtered through a Millipore membrane and analyzed by HPLC-MS.

The organic compounds present in the reaction mixture were extracted with ethyl acetate. The organic layer was dried with anhydrous sodium sulfate. Then, the solvent was removed by evaporation at 40 °C under vacuum. The extract was eventually recovered and stored in amber glass vials to avoid any effect of sunlight or room light lamps. The resulting organic samples were analyzed by FT-IR, ^1^H-NMR, and direct mass spectrometry. 

Before FT-IR or NMR analysis, the organic compounds were separated by thin layer chromatography (TLC) using commercially available aluminum baked plates (Merck 60-F264) as adsorbent material and a mixture of hexane and ethyl acetate as mobile phase. The separated fractions were visualized with an ultra-violet light lamp (245–366 nm). Then, the organic compounds of each fraction were dissolved in a volatile solvent. This solution was transferred dropwise to the surface of a KBr pellet for FT-IR analysis. Another solution was placed in an amber vial to evaporate the solvent before ^1^H-NMR analysis.

### 3.6. Analytical Methods

The reaction samples of the photodegradation, adsorption, and photocatalytic degradation experiments of naproxen were analyzed by UV–Vis spectroscopy in a Shimadzu UV-2600 PC spectrophotometer using standard quartz cells. Although naproxen presents a strong π–π* transition at 230 nm, the scan was performed from 190 to 800 nm. High-performance liquid chromatography (HPLC) analysis of the reaction samples was carried out in a Thermo Scientific Surveyor instrument equipped with a photodiode array UV–Vis detector. A Zorbax Eclipse XDB-C-18 column (4.6 mm × 150 mm × 3.5 μm) was used to separate undegraded naproxen and organic reaction products. The mobile phase consisted of a mixture of acetonitrile and acidified water with 3% glacial acetic acid (50/50 *v*/*v*). The eluent was delivered at a rate of 1.0 mL min^−1^ in isocratic mode operation and the sample volume was 10 μL. Under these experimental conditions, naproxen is eluted around 4.35 min. Some of the reaction samples were also analyzed using an Agilent Infinity II 1260 Prime LC System coupled to a triple quadrupole mass detector. Total organic carbon (TOC) in these samples was measured with a Shimadzu carbon analyzer model 5000A. 

Infrared spectra of the organic extract of the reaction samples were obtained in a Nicolet model iS10 Thermo Scientific FT-IR spectrometer equipped with a diamond ATR cell. The organic extract of the reaction samples was also analyzed in a Bruker 400 MHz ^1^H-NMR spectrometer using CDCl_3_ as solvent. All ^1^H-NMR shifts were reported relative to TMS as external standard. To confirm the molecular structure of the main intermediate reaction products, the organic extract was analyzed by ESI-MS by direct injection of the samples into a JEOL Accu-TOFF-DART mass spectrometer.

## 4. Conclusions

Under experimental reaction conditions of this study (pH = 6.6 ± 0.1) both species, catalyst’s surface and organic molecules, are negatively charged. As a consequence, only a small fraction of the medication interacts with the catalyst’s surface and less than 10% of the solute is adsorbed on commercial TiO_2_. Photolysis experiments of naproxen aqueous solutions with UV-A light indicate that pure photochemical reactions play an important role in the photocatalytic degradation of naproxen. A low-concentration NPX solution is fully photodegraded in 3 h of reaction. But only around 18% of NPX is mineralized in 6 h of reaction. The remaining 82% of the medication is transformed to several intermediate organic reaction products. When the reaction is carried out in the presence of TiO_2_, most of the intermediate organic products are also mineralized into CO_2_ and water by the reactive oxidation species (ROS) generated on the surface of the catalyst.

Results of the chemical analysis indicate that photolysis leads to decarboxylation of NPX with the formation of an anion, which in turn is transformed into 1-(6-methoxynaphthalen-2-yl)ethan-1-one MACN and 1-(6-methoxynaphthalen-2-yl)ethan-1-ol (MNETOH). Therefore, at the beginning of any NPX photocatalytic degradation experiment, the reaction takes place by a combination of photochemical and photocatalytic reactions following a series–parallel mechanism. Photolysis of NPX with low-energy radiation induces the formation of an alcohol and a ketone that under photocatalytic reaction conditions are eventually transformed into several naphthalene derivatives. These aromatic compounds are eventually transformed into several polyhydroxylated aromatic compounds that are strongly adsorbed onto the TiO_2_ surface and as a consequence they are quickly oxidized into low-molecular-weight carboxylic acids by an electron transfer mechanism. Then, all the organic molecules present in the reaction slurry are mineralized to CO_2_ and water.

## Figures and Tables

**Table 1:** Diclofenac, ketoprofen, and naproxen Langmuir adsorption parameters.

NSAID	MW g mol^−1^	pH of the Slurry	q_m_ mMol g^−1^	K_eq_ mMol^−1^
Naproxen sodium salt	252.24	6.6 ± 0.1	0.0362	1.4707
Ketoprofen	254.28	6.3 ± 0.3	0.0672	1.7710
Diclofenac sodium salt	318.13	5.8 ± 0.1	0.04013	21.1401

**Table 2:** Intermediates detected by ^1^H-NMR.

Compound	Signals Observed in the ^1^H NMR Spectra
NPX 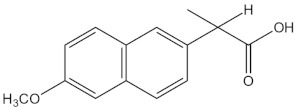	d (ppm): 7.71 (d, 1H), 7.69 (d, 1H), 7.68 (s, 1H), 7.4 (dd, H), 7.14 (dd, 1H), 7.1 (d, 1H), 3.9 (s, 3H OCH_3_), 3.88 (q, 1H), 1.59 (d, 3H, CH_3_)
MACN ketone 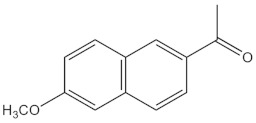	d (ppm): 8.4 (s, 1H), 8.0 (dd, 1H), 7.86 (d, 1H), 7.77 (d, 1H), 7.2 (dd, 1 H), 7.16 (d, 1H), 3.95 (s, 3H OCH_3_), 2.7 (s, 3H, CH_3_)
MNETOH alcohol 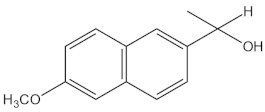	d (ppm): 7.74 (d, 1H), 7.73 (s, 1H), 7.72 (d, 1H), 7.15 (dd, 1H), 7.13 (d, 1H), 5.039 (q, 1H), 3.92 (s, 3H, OCH_3_), 1.57 (d, 3H, CH_3_)
HPA acid 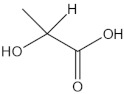	d (ppm): 12.86 (s, 1H, COOH), 4.7 (s, 1H) 4.3 (q, 1H), 1.38 (d, 3H, CH_3_)

**Table 3:** Intermediates detected by HPLC-MS.

Organic Compound	Retention Time (min)	MS Fragmentation Pattern
NPX	4.87	M + 1 = 231, 217, 199, 173, 155
MACN	7.35	M + 1 = 201, 186, 157, 114, 99
MALN	4.1	M + 1 = 186, 185, 170, 158, 114, 99
MHON	5.6	M + 1 = 175, 158, 156, 143

**Table 4:** Intermediates detected by MS in reaction mixtures at different times.

Organic Compound	Irradiation Time (min)	MS of Compound
NPX	90, 120, 180	M + 1 = 232
HONPX	90, 120, 180	M + 1 = 217
MACN	90, 120, 180	M + 1 = 201
MALN	90, 120, 180	M + 1 = 186
MHON	90, 120, 180	M + 1 = 164
THON	120, 180	M + 1 = 192

**Table 5:** Intermediates detected in NPX photochemical or photocatalytic degradation.

Chemical Structure	Compound	MW	Analytical Technique	Ref
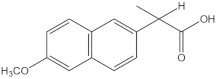	NPX	230	HPLC-MS, GC/MS, NMR	This study, [19,37,40,41,54,56]
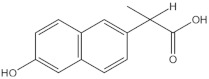	HONPX	216	HPLC-MS, GC/MS	This study, [19,37,41,54,56]
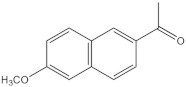	MACN	200	HPLC-MS, GC/MS, NMR	This study, [14,18,22,40,41,54]
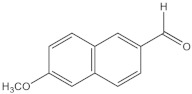	MALN	186	HPLC-MS, GC/MS	This study
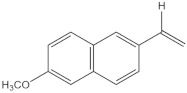	MVN	184	HPLC-MS, NMR	[14,18,19,37,39,41,54]
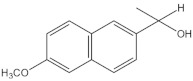	MNETOH	202	HPLC-MS, NMR	[14,18,22,37,40,54]
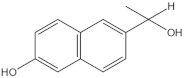	HONETOH	184	HPLC-MS	[19]
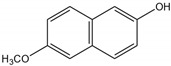	MHON	174	HPLC-MS, GC/MS	This study
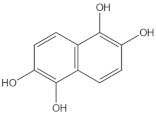	THON	192	GC/MS	This study
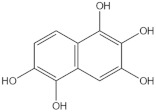	PHON	208	HPLC-MS	[41]
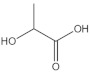	HPA	90	NMR	This study
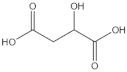	HOSA	134	HPLC-MS	[14]

## Data Availability

Further information about the experimental results is presented in the Supplementary Materials section.

## Supplementary Materials

The following supporting information can be downloaded at: https://www.mdpi.com/article/10.3390/molecules29112583/s1, List of abbreviations; Figure S1: Naproxen (pK_a_ = 4.19) speciation diagram determined by the mass law equation; Figure S2: TiO_2_ point of zero charge determined by the acid–base titration method. Lara-Pérez, C.; Leyva, E.; Zermeño, B.; Osorio, I.; Montalvo, C.; Moctezuma, E. Photocatalytic Degradation of Diclofenac Sodium Salt: Adsorption and Reaction Kinetic Studies. *Environ. Earth Sci.* **2020**, *79*, doi:10.1007/s12665-020-09017-z [31]; Figure S3: Naproxen, ketoprofen, and diclofenac Langmuir adsorption isotherms (TiO_2_ Evonik P25); Figure S4: HPLC chromatogram of a diluted sample of a photocatalytic degradation experiment: (a) naproxen solution, (b) sample after 90 min of reaction, (c) sample after 120 min of reaction, and (d) sample after 180 min of reaction. (Initial concentration = 200 ppm = 0.793 mM, initial pH = 6.4, V = 300 mL, TiO_2_ = 2 g L^−1^, four UV lamps λ_max_ = 365 nm, O_2_ flow = 100 mL min^−1^, reaction time = 90 min, dilution factor for HPLC analysis = 1:10); Figure S5: Photocatalytic oxidation of naproxen monitored by FT-IR, samples were taken after (a) naproxen spectra, (b) 30 min of reaction, (c) 60 min of reaction, (d) 90 min of reaction, and 180 min of reaction. (Initial concentration = 100 ppm = 0.396 mM, initial pH = 6.8, V = 300 mL, TiO_2_ = 2 g L^−1^, four UV lamps λ_max_= 365 nm, O_2_ flow = 100 mL min^−1^); Figure S6: FT-IR spectra of the TLC fractions of the organic extract of a sample taken after 60 min of reaction (a) naproxen spectra, (b) most polar fraction 1, (c) fraction 2, (d) fraction 3, and (e) least polar fraction 4. (Initial concentration = 100 ppm = 0.396 mM, initial pH = 6.8, V = 300 mL, TiO_2_ = 2 g L^−1^, four UV lamps λ_max_ = 365 nm, O_2_ flow = 100 mL min^−1^); Figure S7: Photocatalytic oxidation of naproxen monitored by NMR. Samples were taken 90, 120, and 180 min of reaction. (Initial concentration = 200 ppm = 0.793 mM, initial pH = 6.8, V = 300 mL, TiO_2_ = 2 g L^−1^, four UV lamps λ_max_ = 365 nm, O_2_ flow = 100 mL min^−1^); Table S1: Material balance of the photochemical degradation of a naproxen solution, Figure 1 data; Table S2: Material balance of the photocatalytic degradation of a naproxen solution, Figure 2 data.
