# Supplementary material for: Photocatalytic Degradation of Naproxen: Intermediates and Total Reaction Mechanism
Source: Molecules. 2024 May 30;29(11):2583. doi: 10.3390/molecules29112583 (PMC11174131; doi:10.3390/molecules29112583)
Supplement: Supplementary file 1 [file molecules-29-02583-s001.zip › molecules-3012906-supplementary.pdf]

Photocatalytic degradation of naproxen: Intermediates and total reaction mechanism

Daniela González-Pereyra [1], Ilse Acosta [1], Brenda Zermeño[1], Johana Aguilar[1], Elisa Leyva[*], Edgar Moctezuma[*]

[1] Facultad de Ciencias Químicas, Universidad Autónoma de San Luis Potosí, Av. Manuel Nava # 6, San Luis Potosí, S.L.P., México 78210, danni.gper@gmail.com ORCID: 0000-0002-7098-2359 (D.G.P); acosta.m_ilse@hotmail.com ORCID: 0000-0002-4891-2899 (I.A.); brenda.zermeno@uaslp.mx ORCID: 0000-0003-0958-6450 (B.Z.); johana.aguilar@uaslp.mx ORCID: 0000-0002-7994-3516 (J.A).

* Correspondence: elisa@uaslp.mx ORCID: 0000-0003-3281-2460 (E.L), edgar@uaslp.mx, ORCID: 0000-0002-6350-3786 (E.M.)

List of abbreviations

A^-	Conjugated base
AOP	Advanced oxidation processes
ATR	Attenuated total reflectance
BOD	Biological Oxygen Demand
C_{eq}	Naproxen concentration in the aqueous solution at equilibrium (mMol L^{-1})
COD	Chemical oxygen demand
DHON	naphthalene-2,6-diol
e_{cb}^-	Conduction band electron
ESI-MS	Electro spray ionization mass spectrometry
FT-IR	Fourier Transform Infrared Spectroscopy
GC/MS	Gas chromatography–mass spectrometry
H^+	Hydrogen ion
HA	Undissociated acid
^{1}H-NMR	Proton nuclear magnetic resonance
$HO^\bullet$	Hydroxyl radical
$HO_2^\bullet$	Hydroperoxyl radical
HONETOH	6-(1-hydroxyethyl)naphthalen-2-ol
HONPX	2-(6-hydroxynaphthalen-2-yl) propanoic acid
HOSA	2-hydroxysuccinic acid
HPA	2-hydroxypropanoic acid
HPLC	High-performance liquid chromatograph
HPLC-MS	High-performance liquid chromatograph-mass spectrometry
h_{vb}^+	Valence band positive hole
k_a	Acid dissociation constant
K_{eq}	Langmuir isotherm constant ($mM^{-1}L$)
MACN	1-(6-methoxynaphthalen-2-yl)ethan-1-one
MALN	6-methoxy-2-naphthaldehyde
MHON	6-methoxynaphthalen-2-ol
MNETOH	1-(6-methoxy-2-naphthyl) ethanol or 1-(6-methoxynaphthalen-2-yl)ethan-1-ol
MVN	2-methoxy-6-vinylnaphthalene
NPX	Naproxen or 2-(6-methoxynaphthalen-2-yl)propanoic acid
NSAID	Non-steroidal anti-inflammatory drugs
$O_2^{\bullet -}$	Superoxide radical anion
pH	Logarithm of the reciprocal of the hydrogen ion activity
PHON	naphthalene-1,2,3,5,6-pentanol
pK_a	Logarithmic of the acid dissociation constant
PZC	Point zero charge
q_m	Maximum uptake of NPX per gram of catalyst (mMol g^{-1})
q_s	Uptake of NPX adsorbed on the surface (mMol g^{-1})

R	Radical
ROS	Reactive oxidation species
THON	tetrahydroxy naphthalene
TLC	Thin layer chromatography
TMS	Tetramethylsilane
TOC	Total Organic Carbon
UV-A	Long-wave UV radiation (315–400 nm)
UV-C	Short-wave UV radiation (200-280 nm)

Supplementary material

Top Catal (2013) 56:1875–1882
DOI 10.1007/s11244-013-0119-x

ORIGINAL PAPER

Photocatalytic Degradation of Metoprolol Tartrate

Edgar Moctezuma · Elisa Leyva · Mariana López · Alfonso Pinedo · Brenda Zermeño · Benito Serrano

Since the concentration of all the organic intermediate reaction products for the photocatalytic degradation of metoprolol tartrate can not be easily determined, it is convenient to determine the fraction of the original reactant transformed to intermediate organic products by a material balance based on HPLC and TOC analysis [21, 28]. First, the relative dimensionless metoprolol tartrate and TOC concentrations are calculated using the following equations:

$$aC_m = \frac{C_m}{C_{m0}} \quad (4)$$

$$aTOC = \frac{TOC}{TOC_0} \quad (5)$$

The fraction of metoprolol tartrate mineralized to CO_2 is determined with Eq. 6.

$$fCO_2 = 1 - aTOC \quad (6)$$

Finally, the fraction of metoprolol tartrate transformed to intermediate organic products is calculated with Eq. 7.

$$fPOI = aTOC - aC_m \quad (7)$$

Table S1. Material balance of the photochemical degradation of a naproxen solution Figure 1 data

Material balance of the photochemical degradation of a naproxen solution Figure 1 data							
t	TOC [C/Co]	t	HPLC [A/Ao]	t	Fractio POI	t	Mineralization
0	1	0	1	0	0	0	0
15	0.99180887	15	0.9221261	15	0.06968278	15	0.008191126
30	0.98430034	30	0.78131813	30	0.20298221	30	0.015699659
45	0.99249147	45	0.80483229	45	0.18765918	45	0.007508532
60	0.98703072	60	0.55381372	60	0.43321699	60	0.012969283
90	0.93720137	90	0.34464899	90	0.59255237	90	0.062798635
120	0.92559727	120	0.23642678	120	0.68917049	120	0.07440273
180	0.9221843	180	0.24421869	180	0.67796561	180	0.0778157
240	0.91604096	240	0.0716704	240	0.84437056	240	0.083959044
300	0.85460751	300	0	300	0.85460751	300	0.145392491
360	0.81706485	360	0	360	0.81706485	360	0.182935154

Table S2. Material balance of the photocatalytic degradation of a naproxen solution Figure 2 data

Material balance of the photocatalytic degradation of a naproxen solution Figure 2 data						
t	aTOC [C/Co]	t	aHPLC [A/Ao	t	t	POI=aTOC-aHPLC
-60	1	-60	1	-60	-60	0
0	0.83575925	0	0.67911696	0	0	0.156642289
15	0.71970889	15	0.0224548	15	15	0.697254096
30	0.63060582	30	0.01221217	30	30	0.61839365
45	0.54616444	45	0.01336677	45	45	0.53279767
60	0.47128246	60	0.00949973	60	60	0.461782726
90	0.31437844	90		90	90	0.314378442
120	0.29510228	120		120	120	0.295102282
180	0.17118411	180		180	180	0.171184107
240	0.13607396	240		240	240	0.136073958
300	0.1633753	300		300	300	0.163375295
360	0.17344611	360		360	360	0.173446105

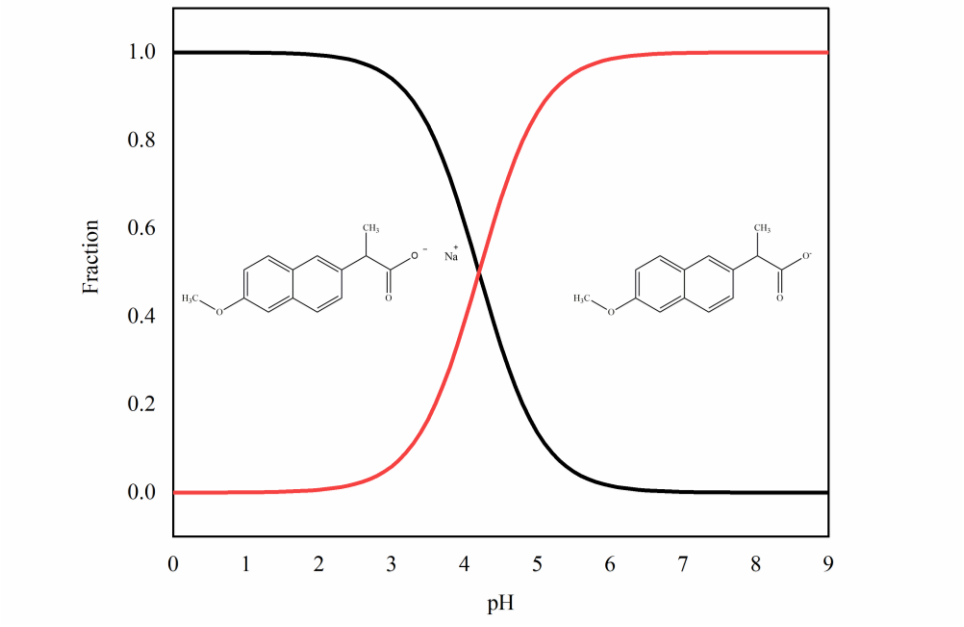

Figure S1. Naproxen (pKa=4.19) speciation diagram determined by the mass law equation

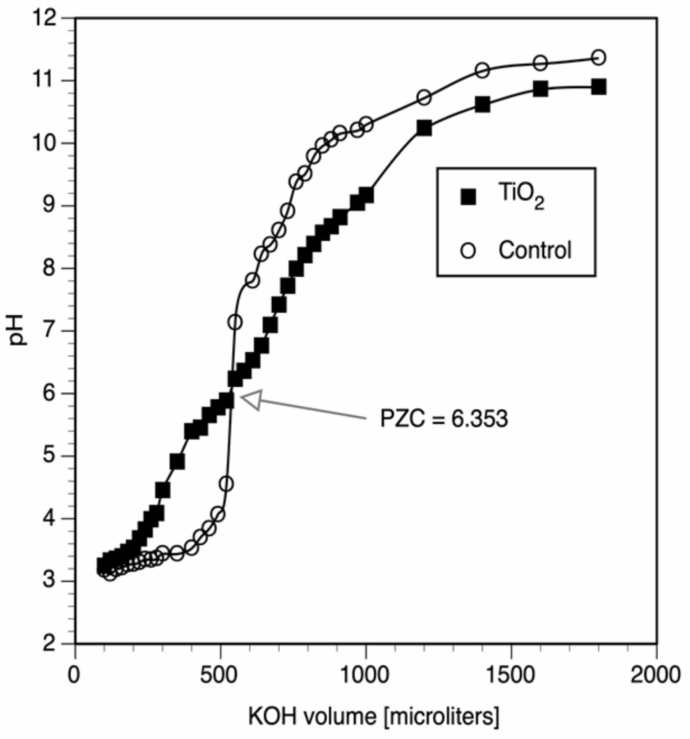

Figure S2. TiO_2 point of zero charge determined by the acid–base titration method Lara-Pérez, C.; Leyva, E.; Zermeño, B.; Osorio, I.; Montalvo, C.; Moctezuma, E. Photocatalytic Degradation of Diclofenac Sodium Salt: Adsorption and Reaction Kinetic Studies. *Environ Earth Sci* 2020, *79*, doi:10.1007/s12665-020-09017-z.

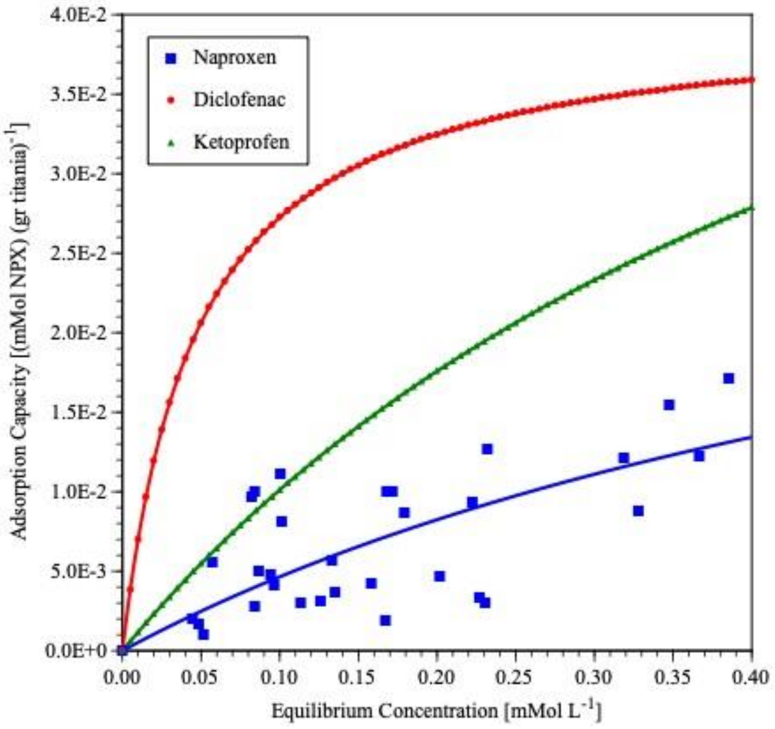

Figure S3. Naproxen, ketoprofen and diclofenac Langmuir adsorption isotherms (TiO_2 Evonik P25)

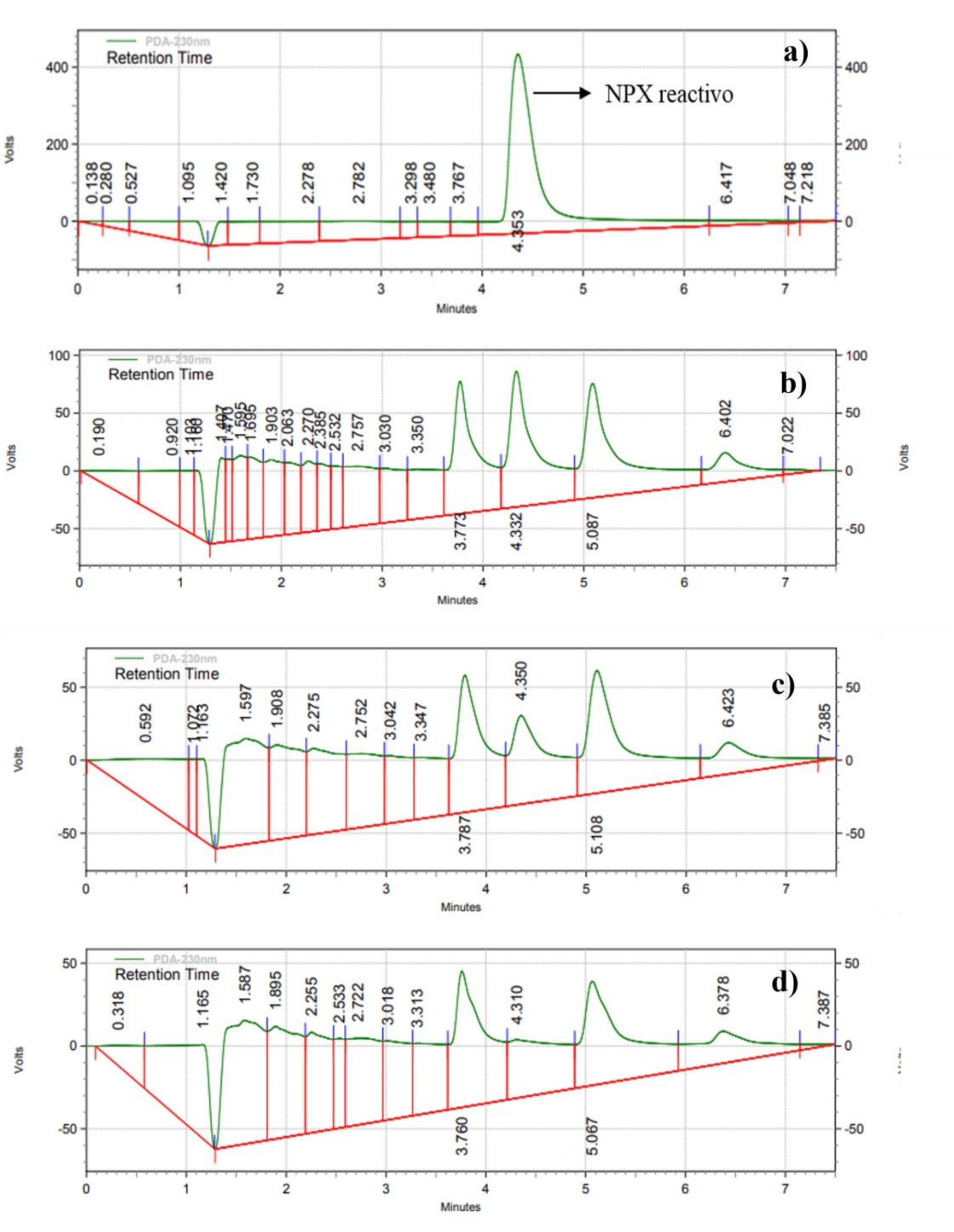

Figure S4. HPLC chromatogram of a diluted sample of a photocatalytic degradation experiment: a) naproxen solution, b) sample after 90 minutes of reaction, c) sample after 120 minutes of reaction and d) sample after 180 minutes of reaction .(Initial concentration = 200 ppm = 0.79 mM, Initial pH = 6.4, V = 300 mL, TiO_2 = 2 g L^{-1}, four UV lamps λ_{max}= 365 nm, O_2 flow = 100 mL min^{-1}, reaction time = 90 minutes, dilution factor for HPLC analysis = 1:10)

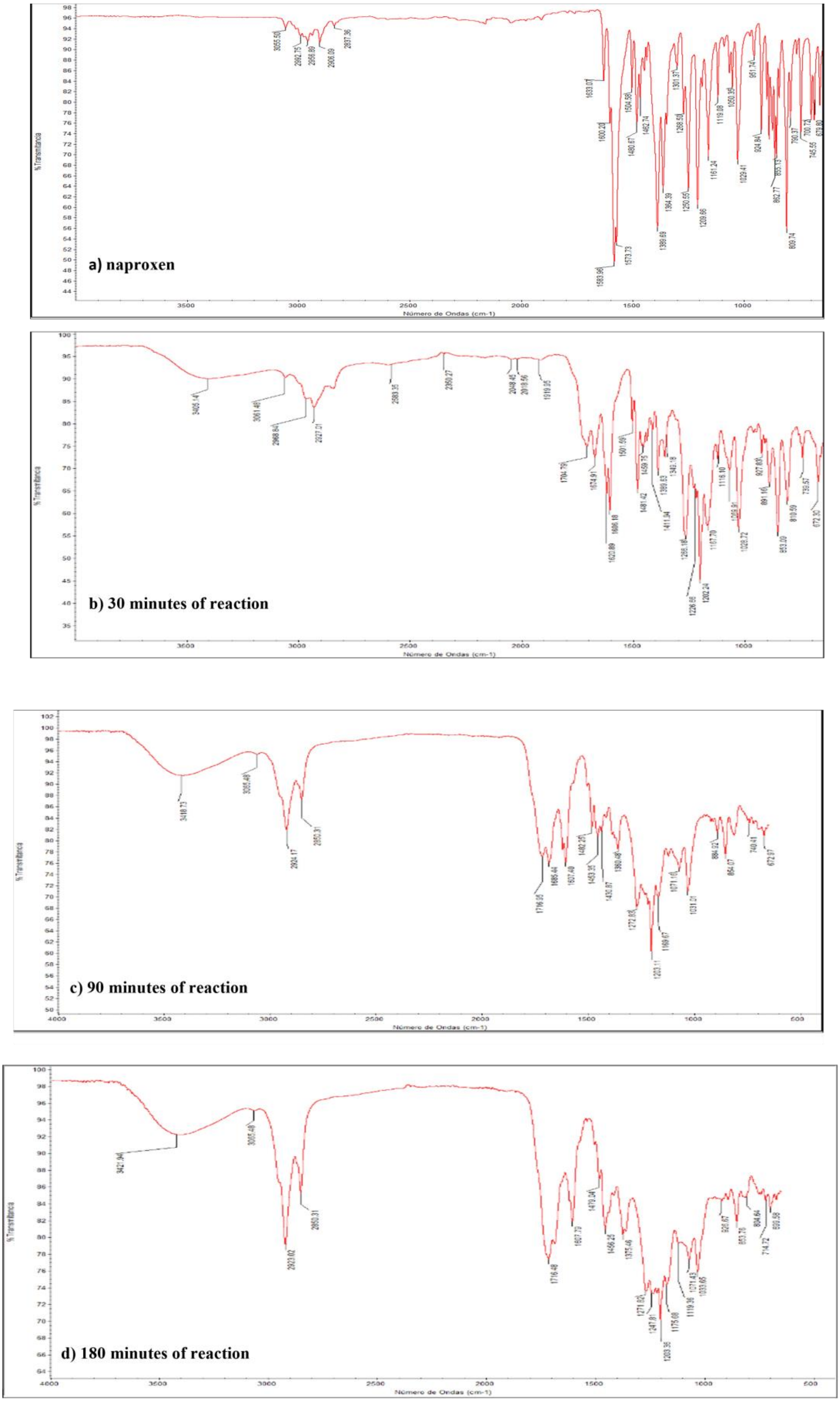

Figure S5. Photocatalytic oxidation of naproxen monitored by FT-IR, Samples were taken after a) naproxen spectra, b) 30 minutes of reaction, c) 60 minutes of reaction, d) 90 minutes of reaction and 180 minutes of reaction. (Initial concentration = 100 ppm = 0.79 mM, Initial pH = 6.8, V = 300 mL, TiO_2 = 2 g L^{-1}, four UV lamps λ_{max}= 365 nm, O_2 flow = 100 mL min^{-1})

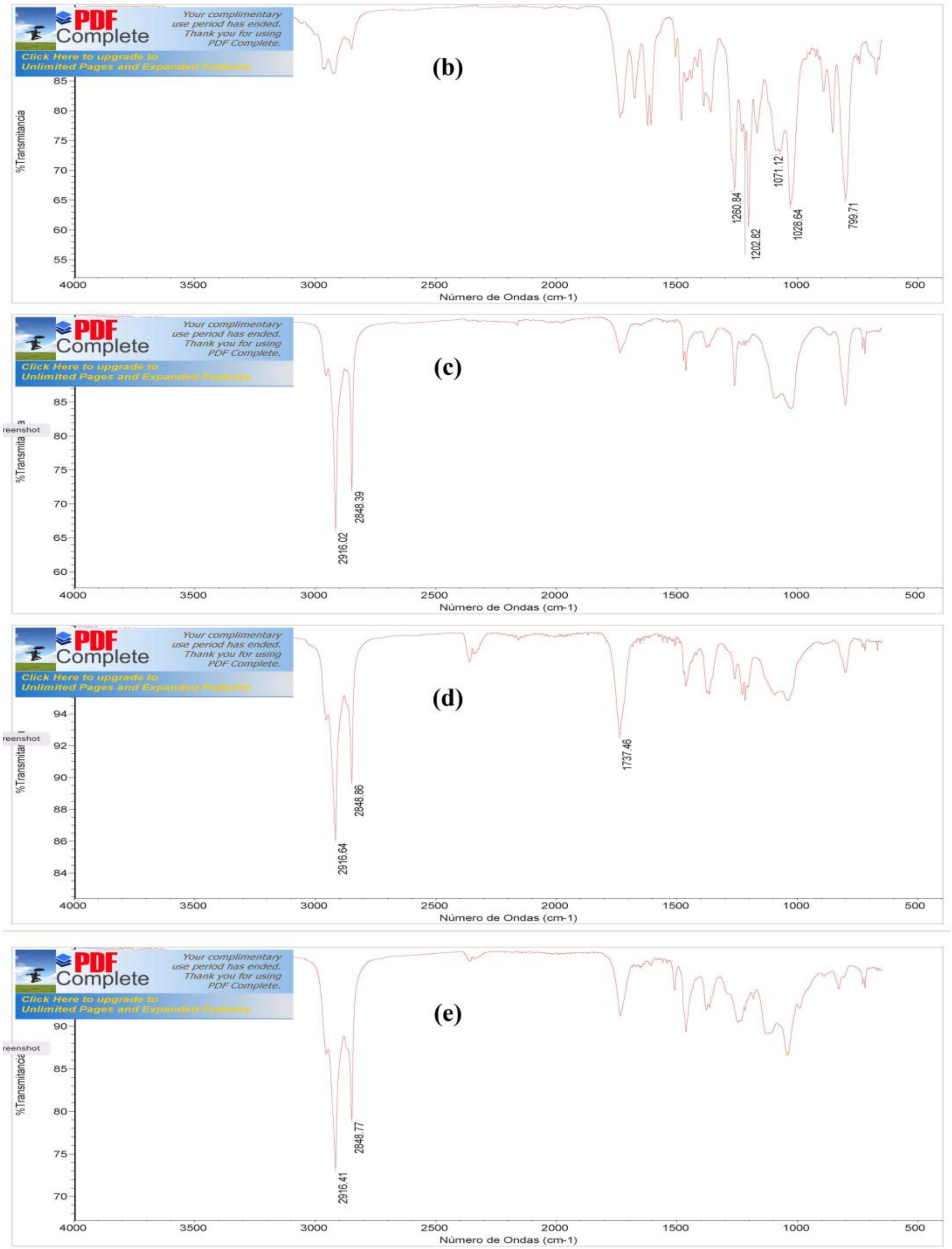

Figure S6. FT-IR spectra of the TLC fractions of the organic extract of a sample taken after 60 minutes of reaction a) naproxen spectra, b) most polar fraction 1, c) fraction 2, d) fraction 3 and 4) least polar fraction. (Initial concentration = 100 ppm = 0.79 mM, Initial pH = 6.8, V = 300 mL, TiO_2 = 2 g L^{-1}, four UV lamps λ_{max}= 365 nm, O_2 flow = 100 mL min^{-1})

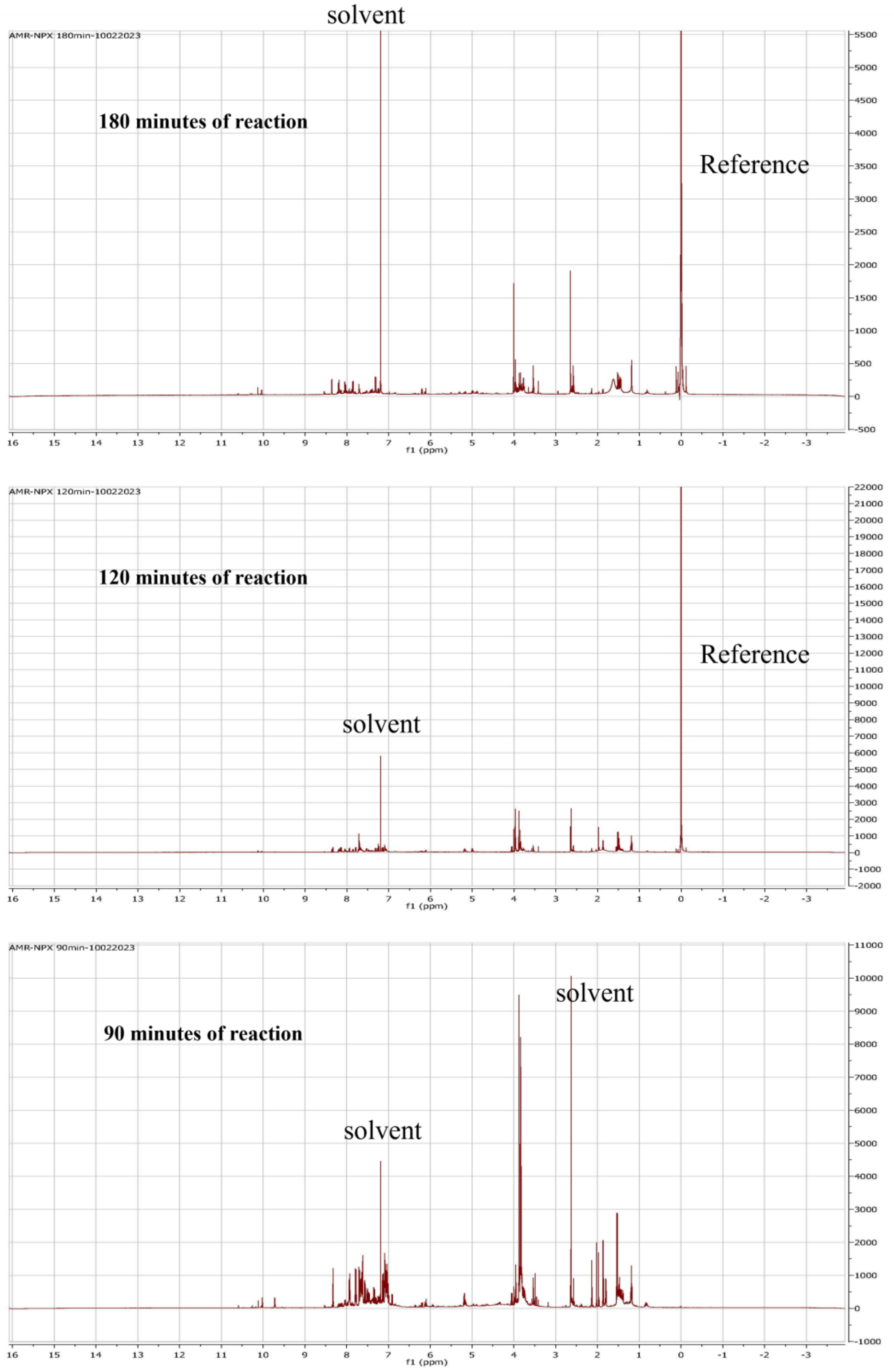

Figure S7. Photocatalytic oxidation of naproxen monitored by NMR. Samples were taken 90, 120 and 180 minutes of reaction. (Initial concentration = 200 ppm = 1.58 mM, Initial pH = 6.8, V = 300 mL, TiO_2 = 2 g L^{-1}, four UV lamps λ_{max}= 365 nm, O_2 flow = 100 mL min^{-1}).
